# Detection of Atrial Fibrillation in Holter ECG Recordings by ECHOView Images: A Deep Transfer Learning Study †

**DOI:** 10.3390/diagnostics15070865

**Published:** 2025-03-28

**Authors:** Vessela Krasteva, Todor Stoyanov, Stefan Naydenov, Ramun Schmid, Irena Jekova

**Affiliations:** 1 Institute of Biophysics and Biomedical Engineering, Bulgarian Academy of Sciences, Acad. G. Bonchev Str. Bl. 105, 1113 Sofia, Bulgaria; vessika@biomed.bas.bg (V.K.); todor@biomed.bas.bg (T.S.); 2 Department of Internal Diseases “Prof. St. Kirkovich”, Medical University of Sofia, 1431 Sofia, Bulgaria; snaydenov@gmail.com; 3 Signal Processing, Schiller AG, Altgasse 68, CH-6341 Baar, Switzerland; ramun.schmid@schiller.ch

**Keywords:** Holter ECG monitoring, deep neural networks, ImageNet DNN, arrhythmia, ECG signal processing, image processing, retraining, fine-tuning, electrocardiomatrix

## Abstract

**Background/Objectives**: The timely and accurate detection of atrial fibrillation (AF) is critical from a clinical perspective. Detecting short or transient AF events is challenging in 24–72 h Holter ECG recordings, especially when symptoms are infrequent. This study aims to explore the potential of deep transfer learning with ImageNet deep neural networks (DNNs) to improve the interpretation of short-term ECHOView images for the presence of AF. **Methods**: Thirty-second ECHOView images, composed of stacked heartbeat amplitudes, were rescaled to fit the input of 18 pretrained ImageNet DNNs with the top layers modified for binary classification (AF, non-AF). Transfer learning provided both retrained DNNs by training only the top layers (513–2048 trainable parameters) and fine-tuned DNNs by slowly training retrained DNNs (0.38–23.48 M parameters). **Results**: Transfer learning used 13,536 training and 6624 validation samples from the two leads in the IRIDIA-AF Holter ECG database, evenly split between AF and non-AF cases. The top-ranked DNNs evaluated on 11,400 test samples from independent records are the retrained EfficientNetV2B1 (96.3% accuracy with minimal inter-patient (1%) and inter-lead (0.3%) drops), and fine-tuned EfficientNetV2B1 and DenseNet-121, -169, -201 (97.2–97.6% accuracy with inter-patient (1.4–1.6%) and inter-lead (0.5–1.2%) drops). These models can process shorter ECG episodes with a tolerable accuracy drop of up to 0.6% for 20 s and 4–15% for 10 s. Case studies present the GradCAM heatmaps of retrained EfficientNetV2B1 overlaid on raw ECG and ECHOView images to illustrate model interpretability. **Conclusions**: In an extended deep transfer learning study, we validate that ImageNet DNNs applied to short-term ECHOView images through retraining and fine-tuning can significantly enhance automated AF diagnoses. GradCAM heatmaps provide meaningful model interpretability, highlighting ECG regions of interest aligned with cardiologist focus.

## 1. Introduction

### 1.1. Atrial Fibrillation Detection in Holter ECG Recordings: Challenges and Current Practices

Atrial fibrillation (AF) is the most common sustained supraventricular arrhythmia, caused by uncoordinated atrial activity that results in ineffective atrial contractions and an irregular heart rhythm [1]. AF can be associated with serious complications, including stroke, peripheral arterial thromboembolic events, heart failure, cognitive decline, and vascular dementia, all of which increase mortality risk [1,2,3,4]. Additionally, patients with AF often experience a range of symptoms and have a reduced quality of life. With an aging population, the prevalence of AF is expected to double in the coming decades, thereby increasing the impact on health services [1].

Early AF detection and prompt treatment are critical to prevent complications and improve long-term outcomes [1,3,4]. AF is often diagnosed based on typical symptoms and characteristic findings on a standard 12-lead electrocardiogram (ECG). Eminent ECG features include the absence of discernible and regular P waves, the presence of high-frequency small-amplitude fibrillatory (f) waves, irregular ventricular activity, and variable RR interval patterns in the absence of an atrioventricular block. However, diagnosis becomes more challenging in cases of asymptomatic episodes [1].

Long-term ECG monitoring with Holter devices and wearable technologies is recommended for patients with suspected arrhythmias that may be transient and missed on the routine resting 12-lead ECG [1,5,6]. Detecting short-term arrhythmic events, particularly in paroxysmal AF, can be challenging in 24–72 h recordings, especially when symptoms are infrequent. While the minimum duration of AF required for diagnosis on wearable devices is not clearly defined, there is general agreement that episodes lasting 30 s or longer may be sufficient for diagnosis, despite limited supporting evidence.

Accurate ECG interpretation depends on various factors, including the clinician’s expertise, ECG quality, and underlying conditions [1,4,6]. Differentiating AF from rhythms that can mimic AF, such as atrial flutter, multifocal atrial tachycardia, or frequent premature atrial contractions, requires expert interpretation and, in some cases, supplemental diagnostic tests like echocardiography or electrophysiological studies [1,2,3,4,5,6]. Moreover, patients with AF may present atypical ECG findings due to underlying conditions, such as left bundle branch block, pre-excitation syndromes, or pacemakers, which can mask or alter the basic AF features, like absent P waves and irregularly irregular QRS complexes [1,2,7]. Additionally, motion artifacts, muscle tremors, and baseline wander are factors that strongly affect diagnostic ECG components. Therefore, providing high-quality ECGs through better sensors, digital filtering, or selecting the optimal snapshot time for analysis are effective strategies to improve both visual interpretation and automated AF detection [1,4,6,8]. Automated ECG algorithms, though becoming more technologically advanced, can still produce false positives or negatives, requiring human review. Clinicians’ expertise is another human-dependent factor that introduces variability in interpretation, especially in borderline or ambiguous cases. Interpretation uncertainty can delay diagnosis or lead to incorrect treatment [1,2,3,4,5]. In resource-restricted settings with limited access to long-term monitoring devices, advanced diagnostic tools, or experienced clinicians, there is an increased risk of misdiagnosis or delayed detection of AF [1,3,9].

### 1.2. Advanced Colormap Modality for ECG Interpretation

Clinicians often need to quickly review 24–72 h Holter ECG recordings, relying on visual assessments of ECG data in a compressed format. One solution is the 2D heartbeat colormap, which aligns consecutive heartbeats and represents signal amplitudes using color, making arrhythmia-related abnormalities in ECG wave positions, heights, and durations easily detectable. This advanced 2D ECG transformation was introduced in 2010 as ECHOView™, part of the DARWIN diagnostic tool of Schiller Medilog ECG Holter systems [10]. In 2015, a similar transformation appeared in the literature under the name Electrocardiomatrix colormap [11].

Studies that rely on visual interpretations of ECG colormaps demonstrate their applicability for analysis of heart rate variability [12]; identification of atrial fibrillation and/or atrial flutter [13,14]; recognition of supraventricular arrhythmias (supraventricular extrasystoles, paroxysmal supraventricular arrhythmia, sinus tachycardia, supraventricular tachycardia, atrial fibrillation, and flutter) and ventricular arrhythmias (ventricular extrasystoles, non-sustained ventricular tachycardia) [15]; and detection of congestive heart failure events [16,17]. After 2020, advancements in deep learning for image processing enabled the use of 2D convolutional neural networks (CNNs) for AF detection [18,19,20] and biometric identification [21].

### 1.3. Deep Transfer Learning for ECG Signals

The robust training of deep neural networks (DNNs) requires extensive labeled datasets that are not always available in the case of biomedical physiological signals, such as the ECG. A solution to this problem is found in transfer learning, which is a promising approach to overcome the requirement for large-scale data volumes in the training process [22].

Transfer learning is widely applied in DNNs developed for the ImageNet Large-Scale Visual Recognition Challenge [23]. These networks, trained on over one million natural images from the ImageNet-1K dataset to classify 1000 object categories, have been adapted for ECG classification through retraining and fine-tuning [24,25,26,27,28,29,30,31,32,33,34,35,36,37,38,39,40,41]. Commonly used architectures include VGG [24], VGG16 [25,26,28,29], AlexNet [28,30,31,32,33], ResNet [24], and variants such as ResNet18 [25,31,32,34,35], ResNet34 [36], ResNet50 [25,26,37], ResNet152 [28], as well as SqueezeNet [32,33], ShuffleNet [32], GoogLeNet [25,31,32,33,38,39], DenseNet-121,169,201 [25,26,32,41], InceptionV3 [26,32], EfficientNet [25,26,27], EfficientNetV2 [26], MobileNetV2 [26,40], NASNetMobile [26], Xception [26], etc.

A recent scoping review summarized findings from 70 studies published between 2018 and 2024, focusing on ECG-based transfer learning for cardiovascular disease detection [22]. The range of ECG analysis tasks addressed through transfer training includes heartbeat classification [24,34,42], arrhythmia classification [25,30,31,32,33,36,37,38,39,43], AF detection [26,34,35,40,41,44,45,46], detection of myocardial infarction [27,29,30,37], and ECG analysis during cardiopulmonary resuscitation [47], etc. ECG signal representations used for the DNN input often include time-frequency domain transformations, such as continuous wavelet transforms and scalogram images [25,32,33,36,37,38], or short-time Fourier transforms [34,40]. Additionally, ECG classification models process time-domain ECG images in various formats, such as standard 12-lead ECG plots [41], images capturing temporal, spatial, and amplitude-related information in 2 to 12 ECG leads [39], single-lead ECG traces [28,29], ECHOView colormap representations of heartbeat waveforms in a single lead [26], and raw ECG time-series data [24,30,31,35].

Few studies propose custom DNN models that are typically trained from scratch on large ECG datasets with general annotations or even without labels. These models are then retrained and fine-tuned on smaller target datasets with task-specific labels [42,43,44,45,46,47].

### 1.4. DNN Models Interpretability

Researchers have primarily focused on improving the performance of ECG analysis models while often overlooking issues related to the interpretability and explainability of machine learning and deep learning algorithms [48]. Explainable methods highlight the relevance of specific features or regions in the input time-series samples or images for rhythm classification, typically visualized as saliency maps or heatmaps [49,50,51,52,53]. Among the most commonly used explainable deep learning techniques for AF detection are Gradient-weighted Class Activation Mapping (GradCAM) and its extensions [50,51,53,54,55], local interpretable model-agnostic explanations (LIME) [56,57,58], and Shapley additive explanations (SHAP) [59,60]. Other explainability techniques tailored for AF detection models include the cumulative weight estimation of activated neurons along the decision path [61], hierarchical attention networks [62], and ECG occlusion analysis, which assesses the influence of input features by selectively masking them [63].

A recent study questioned the impact of transfer learning on explanation accuracy, reporting that GradCAM failed to provide meaningful insights and instead focused on ECG regions irrelevant to decision-making [29]. However, this discrepancy has not been observed in other studies that combine transfer learning with different explanation techniques, such as LIME [56,58] and GradCAM [24]. The explainability of transfer-learning DNNs is an open topic for further investigation.

### 1.5. Research Objectives

In this study, we focus on the automatic AF detection in Holter ECG recordings using state-of-the-art interpretation techniques. Specifically, we aim to explore the potential of deep transfer learning with image recognition DNNs to improve the interpretation of novel ECHOView diagnostic images for the presence of AF. To achieve this, we compare the performance of 18 retrained and fine-tuned ImageNet DNN models, evaluating their AF detection accuracy vs. computational cost; robustness across different patients and ECG leads; effect of shortening the analysis duration from 30 s down to 10 s; and noise robustness during continuous Holter monitoring. Finally, we apply GradCAM’s model interpretation capability to visualize the regions of interest within the DNN’s receptive field, linking these to input ECHOView images and corresponding ECG waveforms. Thus, we can compare the DNN-generated decisions with those of cardiologists, highlighting areas of agreement and differences and providing insights into some sources of error.

## 2. Materials and Methods

### 2.1. Holter ECG Database and ECHOView Images

This study uses ECG signals sourced from a new publicly available Holter ECG monitoring database with paroxysmal AF, named the IRIDIA-AF database [64], which includes 152 patients (53.2% male), with a mean age of 72 ± 11 (range 41–99 years) and a mean risk of stroke CHADVASC score in patients with AF 3.16 (range 1–9). The database contains 167 recordings of two-lead ECG signals (I, II) sampled at 200 Hz with a precision of 10 μV over 24–72 h. The database provides annotations for the QRS positions, as well as the onset and offset of AF episodes as annotated and reviewed by expert cardiologists and specialist cardiac nurses [64]. Since the ECG recording bandwidth in the IRIDIA-AF database is not specified, we apply first-order Butterworth pre-filtering to the full-length ECG recordings, aligning the bandwidth with standard monitoring ranges (0.5–40 Hz) [65]. With 6690 h of ECG data, including 1609 h of recorded AF rhythms across different patients and ambulatory conditions, the IRIDIA-AF database is a valuable resource for AF Holter ECG analysis and is considered sufficient for the deep transfer learning in this study.

**Figure 1:**
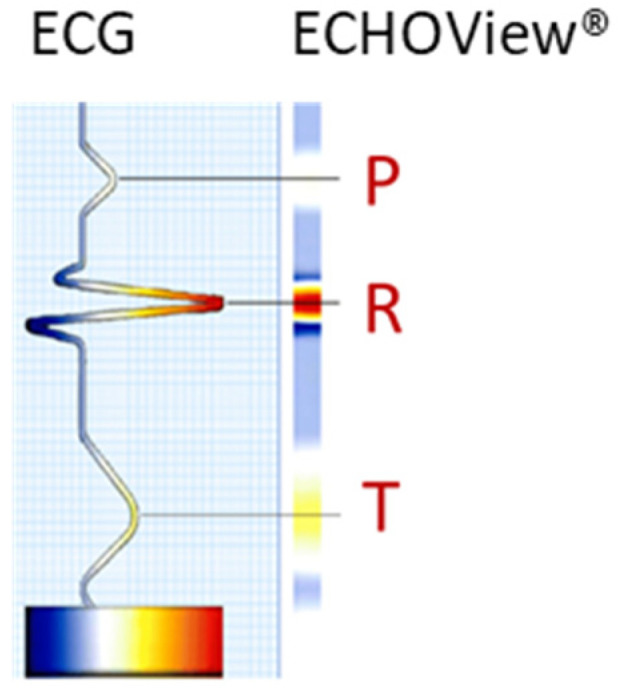
Conversion of an ECG heartbeat to ECHOView™ image using “black-blue-white-orange-red” color code embedded in DARWIN2 software (Schiller AG, Baar, Switzerland).

The ECG signal is transformed to 2D colormaps similar to the ECHOView™ images, which represent a trademark image technology embedded in the “P-wave Detection” analysis module of the Medilog DARWIN2 software (version 2.9, Schiller AG, Baar, Switzerland) for reading and compressed visualization of Holter ECG data [66]. The principle for the transformation of the ECG waveform of a single heartbeat into an amplitude-related colormap is illustrated in Figure 1. Due to the typical amplitude differences between ECG waves, they can be visually distinguished by color. For example, high positive R-peaks appear red, positive T-waves are orange, and low-amplitude P-waves are white. Negative amplitudes, if present, are recognized on a gradient from blue-white to blue-black. Various examples of ECHOView™ images in supraventricular and ventricular arrhythmias can be found in [15].

This study applies a method similar to the ECHOView transform principle illustrated in Figure 1, processing sequential heartbeats over a 30 s analysis interval. Independent images are generated for each ECG lead. Using the generic annotations of QRS positions, the color bars of individual heartbeats are aligned in an ECHOView image with the following dimensions:

- Width: N pixels, corresponding to the number of QRS complexes within the analysis interval, which varies with heart rate.
- Height: 300 pixels (equivalent to 1.5 s at a 200 Hz sampling rate), defining the resolution of the ECHOView segment. This segment represents the heartbeat pattern within a ±0.75 s window around the QRS annotation.

**Figure 2:**
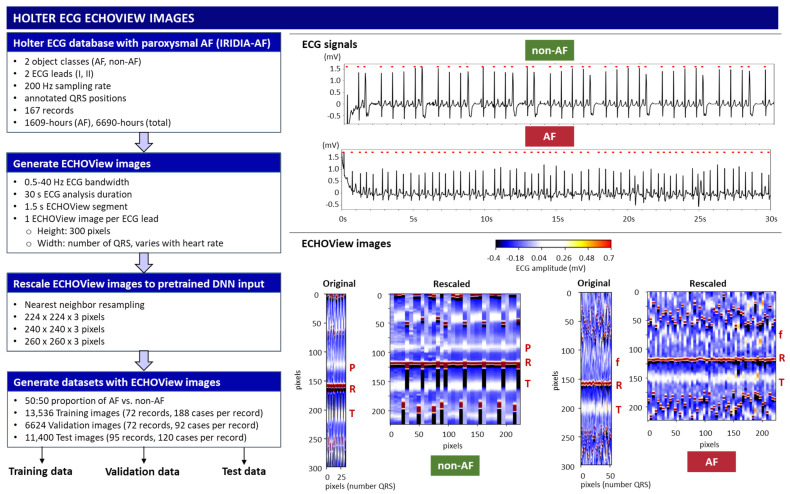
On **left**: Workflow for generating ECHOView images from the Holter ECG IRIDIA-AF database, producing training, validation, and test datasets. On **right**: Illustration of the principle with two examples of 30 s ECG signals (non-AF and AF) with annotated QRS positions (red dots). Below are their corresponding ECHOView images: Original (number QRS × 300 × 3 pixels) and Rescaled (224 × 224 × 3 pixels). R—R-peak of ventricular depolarization (red line); P—P-wave of the normal atrial depolarization (blue-white band); T—T-wave of the ventricular repolarization (blue-white band); f—fibrillation f-waves (white dots).

The flowchart of the method for converting ECG signals into ECHOView images is summarized in Figure 2, illustrated with two representative examples of AF and non-AF rhythms. Due to the higher heart rate in the AF example, its ECHOView image contains more QRS complexes and is therefore wider than the non-AF image. However, fixed image dimensions are required for deep learning input. To ensure compatibility, the original ECHOView images are rescaled to various fixed sizes suitable for ImageNet DNN inputs (224 × 224 × 3, 240 × 240 × 3, 260 × 260 × 3), applying the nearest neighbor image resizing method.

Training, validation, and test datasets with ECHOView images are created by randomly selecting 30 s analysis intervals within the original Holter ECG recordings of the IRIDIA-AF database. The analysis interval is labeled AF or non-AF as defined by the provided manual annotations. The criteria for dataset creation include a balanced 50:50 ratio of AF to non-AF samples selected per record and lead. The training and validation samples are extracted from the first 72 records (000–071) in a ratio of 70:30 per record, including a total of 13,536 training and 6624 validation samples from ECG Leads I and II. The test samples are extracted from the remaining 95 records (072–166), including a total of 11,400 samples, which are independent from the training and validation datasets. The number of cases used in the study is summarized in Table 1.

### 2.2. Deep Transfer Learning

This study investigates deep transfer learning, using large DNNs that were pre-trained on ImageNet-1K—a dataset comprising over 1 million natural images in 1000 object classes originally developed for the Large-Scale Visual Recognition Challenge [23]. From the models integrated into the TensorFlow Keras applications [67], we selected those that offer a trade-off between model complexity, hardware limitations, and training efficiency. The first inclusion criterion is based on the number of trainable parameters, which is limited to fewer than 24 million, excluding the top (classification) layer. Preliminary research indicated that larger training parameters increase computational complexity, and due to hardware limitations, we were forced to use very small batch sizes during training, which may compromise generalization. The second inclusion criterion applies to the input image dimensions. Most small parameter networks also use smaller images, i.e., 224 × 224 × 3 pixels, which is the default input size for models initialized with pre-trained ImageNet weights and biases, such as MobileNetV2 [68], VGG16 [69], NASNetMobile [70], EfficientNetB0 [71], EfficientNetV2B0 [72], InceptionV3 [73], Xception [74], DenseNet121, DenseNet169, DenseNet201 [75], ResNet50, and Resnet50V2 [76]. We also identified other networks from the EfficientNet family [71,72], which have fewer than 24 million parameters but larger input dimensions. These include EfficientNetB1 and EfficientNetV2B1 with 240 × 240 × 3 input, EfficientNetB2 and EfficientNetV2B2 with 260 × 260 × 3 input, EfficientNetB3 and EfficientNetV2B3 with 300 × 300 × 3 input, EfficientNetB4 with 380 × 380 × 3 input, and EfficientNetV2S with 384 × 384 × 3 input. Larger input sizes typically correlate with better model performance. However, to ensure a fair comparison between networks with different architectures, we chose to use input sizes up to 260 pixels for our study. This decision is justified by the fact that the ECHOView images are limited to a maximum of 300 pixels, and using larger images would result in redundant information. Thus, this approach enables a more consistent and meaningful comparison across networks while ensuring a fair evaluation of transfer learning efficiency.

**Figure 3:**
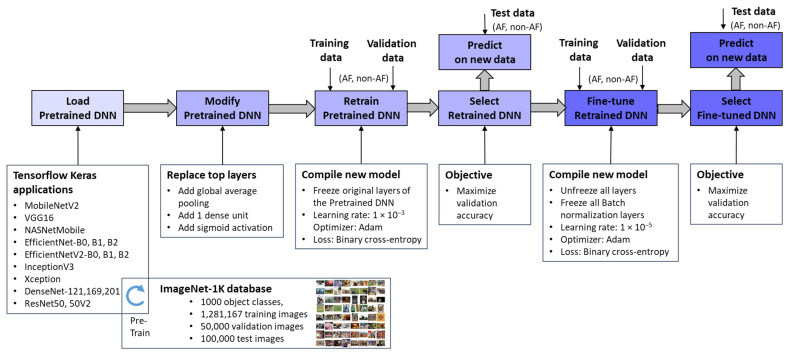
Deep transfer learning applied to retrain and fine-tune specific pretrained ImageNet DNNs for binary arrhythmia classification (AF vs. non-AF) using training, validation, and test data with ECHOView images of Holter ECG.

Figure 3 outlines the workflow for integrating pretrained ImageNet DNNs via TensorFlow Keras applications [67] and adapting them to the new image recognition tasks. Through transfer learning and fine-tuning [77], these models were optimized to process ECHOView images as input, generating a binary classification output for AF detection. It is worth noting that the pixel values of ECHOView images are scaled between 0 and 1, and each channel is normalized with respect to the ImageNet dataset using the <preprocess_input> method specific to each ImageNet DNN, as provided by the TensorFlow Keras applications [67].

The adaptation process involves two key phases:

1. Top Layer Retraining

The pretrained ImageNet DNN architectures were modified by replacing their original classification layers with global average pooling and a single dense layer followed by a sigmoid activation. This transformation converted the 1000-class output into a binary probability for AF detection (pAF ∈ [0, 1]). The modified models were then trained, with only the newly added top layer updated using our training and validation datasets. This process, referred to as “Retrain Pretrained DNN”, ensured that the base layers remain frozen, retaining the knowledge learned from the source domain. Since only a limited number of parameters were adjusted, this approach significantly reduced computational effort compared to training the entire network from scratch, which would be impractical given the limited data available. The retraining phase employed a default learning rate of 1 × 10^−3^ and optimized binary cross-entropy loss. The optimal retrained DNN was selected based on maximizing validation accuracy.

2. Fine-Tuning

The retrained DNNs were further refined through fine-tuning, where all pretrained features (up to 24 million parameters) were gradually adapted to our training data using a very low learning rate of 1 × 10^−5^. While this step offers the potential for enhanced performance on the training set, it also carries a risk of overfitting, particularly with limited data. Therefore, an evaluation of independent Holter recordings is crucial to assess the models’ generalizability. Importantly, batch normalization layers remain frozen during fine-tuning because these layers store running estimates of mean and variance from the original large-scale ImageNet dataset. If batch normalization layers were updated with a small, domain-specific dataset, the new statistics might not be representative, leading to unstable training and poor generalization [77]. By keeping them frozen, the model retains the well-established normalization parameters, ensuring that feature distributions remain consistent and preventing distortions that could arise from limited training data.

### 2.3. Performance Evaluation

Two types of performance metrics are evaluated for each DNN model:

1. Accuracy metrics:

The classification performance reported for the test set is evaluated in terms of true positive rate (TPR), true negative rate (TNR), and accuracy (Acc), applying a threshold policy for detection of the AF-class when the DNN output probability is ≥0.5:

(1) $$TPR=\frac{TP}{TP+FN}\times 100\%,\;TNR=\frac{TN}{TN+FP}\times 100\%,\;Acc=\frac{TP+TN}{TP+TN+FP+FN}\times 100\%,$$

where true positives (TP) and true negatives (TN) represent correctly classified AF and non-AF cases, respectively. False positives (FP) refer to non-AF cases misclassified as AF, while false negatives (FN) denote AF cases incorrectly identified as non-AF.

The area under the receiver operating characteristic curve (AUROC) is another standard metric used to evaluate the performance of a DNN’s probabilistic output across all classification thresholds within the range [0, 1] for distinguishing between AF and non-AF classes. The AUROC score varies from 0 to 1, where 0.5 represents random guessing, and 1 indicates perfect classification performance.

2. Computational cost:

- Inference time ratio: The ratio of a model’s inference time relative to the inference time of the fastest model. It provides a normalized comparison of processing time.
- Number of parameters (weights and biases) in the model: This directly impacts memory usage, computational complexity, and overall efficiency.

### 2.4. ECHOView Image Importance

This study applies the GradCAM technique to generate visual explanations for the decisions of a selected ImageNet DNN model. The approach exploits the spatial information preserved through the convolutional layers by using the target gradients (AF, non-AF) of the final convolutional layer. The resulting heatmap highlights the important regions in the input ECGOView image that are most influential in the classification decision [78,79].

Concerning a feature map $A^{k}$ of the $k^{th}$ filter in the last convolutional layer, the gradients $\alpha^{c}$ of a predicted score $y^{c}$ for class *c* is calculated by the equation:

(2) $$\alpha^{c}=\frac{{dy}^{c}}{{dA}^{k}}$$

The importance value of each filter is computed as the global average pooling of the gradients across the spatial dimensions (*i*, *j*) of $A^{k}$:

(3) $$\alpha_{k}^{c}=\frac{1}{Z}\sum_{i}\sum_{j}\frac{{dy}^{c}}{{dA}_{i,j}^{k}}$$

where *Z* is the total number of pixels in the feature map.

Using the importance values, a weighted combination of the feature maps of all filters (*n*) is calculated:

(4) $$\sum_{k}\alpha_{k}^{c}A^{k}=\alpha_{1}^{c}\cdot A^{1}+\alpha_{2}^{c}\cdot A^{2}+\cdots +\alpha_{n}^{c}\cdot A^{n}$$

Finally, to compute the GradCAM heatmap, a Rectified Linear Unit (ReLU) activation is applied to focus on positive gradients, thus enhancing the most salient features related to the corresponding class:

(5) $$L_{GradCAM}^{c}=ReLU\left(\sum_{k}\alpha_{k}^{c}A^{k}\right)$$

$L_{GradCAM}^{c}$ represents a coarse localization map that has the exact spatial dimensions as the feature maps $A^{k}$. To generate the final heatmap, $L_{GradCAM}^{c}$ is resized to match the dimensions of the input ECHOView image by bilinear interpolation and is then normalized to the range [0, 1].

## 3. Results

### 3.1. Comparative Study of Retrained and Fine-Tuned ImageNet DNNs for AF Detection

ECG signal processing and deep transfer learning were implemented in Python 3.9.5 using Keras and Tensorflow 2.9.1 on a Persy Stinger workstation with Intel CPU Xeon Silver 4214R @ 2.4 GHz (2 processors), 96 GB RAM (Intel, Santa Clara, CA, USA), and NVIDIA RTX A5000-24 GB GPU (NVIDIA, Santa Clara, CA, USA). Eighteen pretrained ImageNet DNNs [68,69,70,71,72,73,74,75,76] were retrained and fine-tuned for up to 450 epochs, using early stopping after validation loss degradation. The hyperparameters of the retraining and fine-tuning phases were set as follows:

- Retraining of the top layer with 513 to 2049 trainable parameters (Table 2) used learning rate = 1 × 10^−3^, optimizer = ‘Adam’, loss function = ‘binary cross-entropy’, batch size = 64.
- Fine-tuning of 0.38 M to 23.5 M trainable parameters (Table 2) used frozen batch normalization layers, learning rate = 1 × 10^−5^, optimizer = ‘Adam’, loss function = ‘binary cross-entropy’, batch size = 32.

The training process curves were shown in a preliminary study published in a conference proceeding [26]. This study provides extended results, comparing the test performance of 18 retrained and 18 fine-tuned ImageNet DNNs, including the following evaluations disclosed in the next sections:

- Test 1: Test accuracy vs. computational cost;
- Test 2: Robustness across different patients;
- Test 3: Robustness across ECG leads;
- Test 4: Effect of shortening the analysis duration;
- Test 5: Noise robustness over full-length Holter ECG recordings.

#### 3.1.1. Test Accuracy vs. Computational Cost

Table 2 summarizes the performance on the full test dataset (Table 1, 11,400 samples from Lead I and Lead II), showing an AF detection accuracy of 92.9–96.3% for retrained DNNs (513–2049 trainable parameters), improving to 96.3–97.6% with fine-tuning (0.38 M–23.48 M parameters). Fine-tuning boosts TPR by up to 6.5% (88.2–93.9% to 93.4–95.8%) while modestly improving TNR by up to 2.8% (96.4–98.7% to 98.9–99.5%). No direct relation is observed between the number of trainable parameters and overall performance.

**Figure 4:**
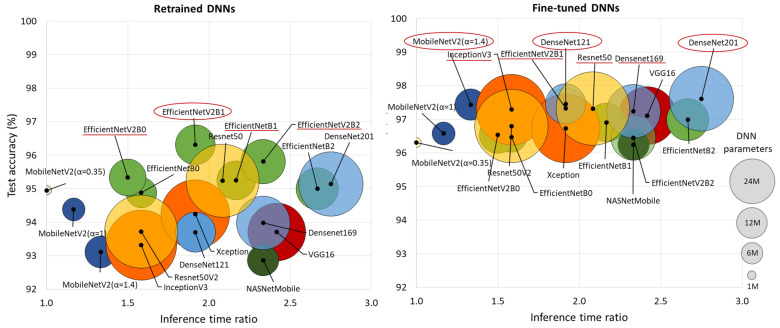
Comparison of retrained and fine-tuned DNNs in respect to their test accuracy for AF detection (y-axis) vs. computational cost, represented by the inference time ratio (x-axis) and DNN parameters (bubble size). The most accurate models are highlighted with red circles (first-ranked) and red lines (second-ranked).

Figure 4 comprehensively compares DNN performance by accuracy and computational cost, which varies by up to 2.75× in inference time and ranges from 0.4 M to 24 M model parameters, highlighting the following models:

- Retrained DNNs: EfficientNetV2B1 (1.92 inference time ratio; 96.3% accuracy) is the most accurate model and has a moderate computational cost. EfficientNetV2B2 (2.33; 95.8%) ranks second but has a higher inference time. EfficientNetV2B0 (1.5; 95.3%) and EfficientNetB1 (2.17; 95.3%) rank third, with a 1% accuracy drop, presenting low and moderate computational costs, respectively.
- Fine-tuned DNNs: MobileNetV2 (α = 1.4) is both fast and highly accurate (1.33; 97.4%). DenseNet121 (1.92; 97.5%) and DenseNet201 (2.75; 97.6%) achieve the highest accuracy but at moderate to high computational costs. InceptionV3 (1.58; 97.3%), EfficientNetV2B1 (1.92; 97.3%), ResNet50 (2.08; 97.3%), and DenseNet169 (2.33; 97.2%) follow closely, balancing strong performance with varying inference times.

#### 3.1.2. Robustness Across Different Patients

The comparison of test versus validation accuracy drop in a DNN model provides insight into its generalization ability, potential overfitting, and robustness across different patients. Generalization is particularly important in ECG analysis, where inter-patient variability can significantly impact model performance. A minimal drop suggests that the model maintains consistent performance on unseen data, indicating strong generalization. Conversely, a significant accuracy drop warns of overfitting, where the model learns patterns specific to the validation set but struggles with new patient data.

**Figure 5:**
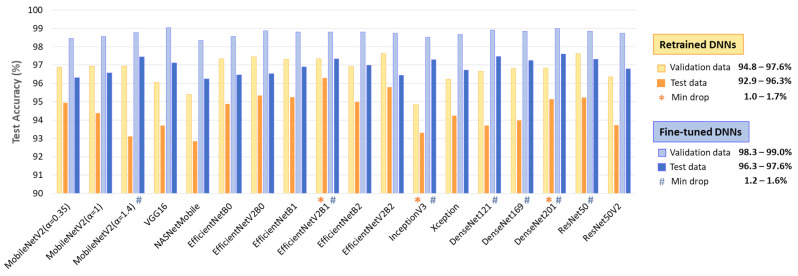
Validation and test accuracy of 18 retrained and fine-tuned DNNs. The top-performing models with minimal test vs. validation accuracy drop are highlighted.

The results in Figure 5 indicate a test vs. validation accuracy drop ranging from 1 to 3.8% for retrained DNNs and 1.2–3.3% for fine-tuned DNNs. Such performance drops are expected in deep learning; however, certain models demonstrate minimal sensitivity to patient-specific variations (drop ≤ 1.7%). Notably, these include retrained EfficientNevV2B1, InceptionV3, DenseNet201, as well as fine-tuned MobileNetV2 (α = 1.4), EfficientNevV2B1, Inception V3, ResNet50, and DenseNet-121, -169, -201.

#### 3.1.3. Robustness Across ECG Leads

Given the varying availability of ECG leads in clinical and ambulatory AF diagnostics, robust performance across different leads is crucial to ensure the model’s reliability and flexibility in real-world wearable applications. This study evaluates Lead I and Lead II, which differ in waveform morphology, polarity, amplitude, and noise levels. A truly robust model should maintain consistent accuracy regardless of the chosen lead. In ECHOView images, these differences transform into distinct colormaps, which may impact the model’s ability to detect AF effectively.

**Figure 6:**
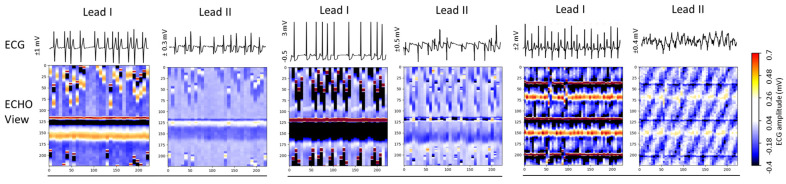
ECG signals (**top**) and corresponding ECHOView images (**bottom**) illustrate the variation in colormaps across Lead I and Lead II of three Holter ECG samples. The amplitude range is indicated on the left of each ECG trace, corresponding to the color bar. For improved waveform visibility, ECG signals are cropped to 10 s, while ECHOView images represent the rhythm in 30 s analysis interval.

Figure 6 presents three Holter ECG samples from the IRIDIA-AF database, highlighting the diversity of colormaps in ECHOView images generated from Lead I and Lead II of the same record. Variations include low contrast in low-amplitude ECGs, saturation in high-amplitude ECGs, and differences in wave coloration, such as T-waves appearing yellow when positive and black when negative, R-waves displaying white for low amplitude and red for high amplitude, or RS-waves exhibiting a red-black pattern. Additionally, some waves are affected by overlapping noise, further illustrating the challenges in ECHOView interpretation.

**Figure 7:**
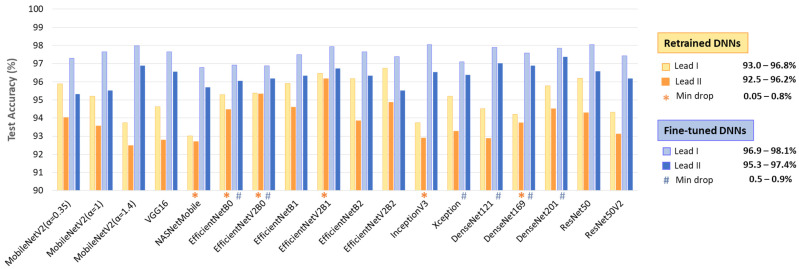
Test accuracies of 18 retrained and fine-tuned DNNs using ECHOView images from Lead I or Lead II. The best models presenting minimal drops in accuracy between leads are highlighted.

The test accuracy results in Figure 7 show that, despite being trained with equal samples from Lead I and Lead II, all DNNs achieve higher accuracy with Lead I. The accuracy drop when using Lead II ranges from 0.05 to 2.3% for retrained DNNs and from 0.5 to 2.1% for fine-tuned DNNs. This performance drop is inversely related to the high informative value of Lead II to capture atrial activity along the heart’s electrical axis and can be attributed to the limited quality of ECHOView images, influenced by lower ECG amplitude and higher noise levels, particularly in Lead II of the IRIDIA-AF database. However, certain models demonstrate minimal sensitivity to lead-specific variations (drop < 1%). Notably, these include retrained NASNetMobile, EfficientNet-B0, -V2B0, -V2B1, Inception V3, DenseNet169, as well as fine-tuned EfficientNet-B0, -V2B0, Xception, DenseNet-121, -169, -201.

#### 3.1.4. Effect of Shortening the Analysis Duration

**Figure 8:**
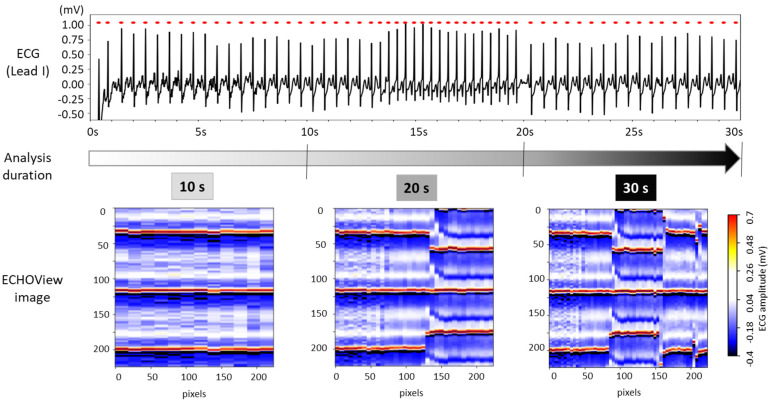
The 30 s ECG trace (**top**) and corresponding ECHOView images (**bottom**) generated for analysis intervals of 10, 20, and 30 s.

The choice of ECG processing interval duration involves a trade-off between accuracy and decision delay. As illustrated in Figure 8, longer intervals capture more information on rhythm evolution, enhancing reliability but introducing latency. In contrast, shorter intervals allow for faster decisions but may be less reliable due to their limited representation of the ECG rhythm.

For this test, ECHOView images were generated for 10, 20, and 30 s intervals while maintaining a consistent colormap. Although the image width varies based on the number of QRS complexes within the analysis interval, all ECHOView images are resized to match the DNN input requirements, as outlined in the flowchart in Figure 2. In Figure 8, the only noticeable difference between the 10, 20, and 30 s ECHOView images is the x-axis granularity (the level of detail or resolution along the x-axis), which is determined by the number of included QRS complexes. The shortest interval (10 s) contains fewer QRS complexes, making individual beats appear wider when the image is rescaled. In contrast, the longest interval (30 s) includes more QRS complexes, leading to finer granularity, where individual beats are more compressed but provide a broader view of rhythm variations over time.

**Figure 9:**
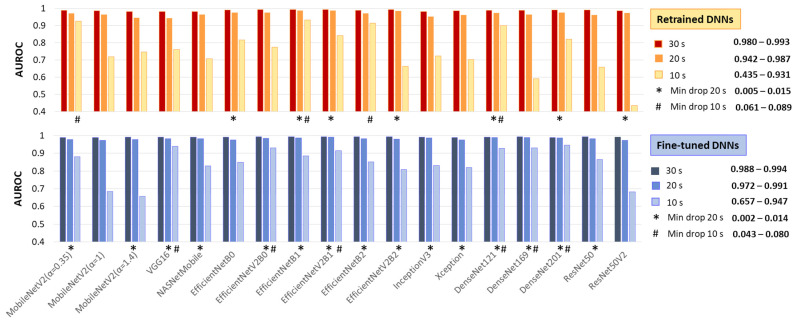
Test AUROC of 18 retrained and fine-tuned DNNs using ECHOView images generated for analysis intervals of 10, 20, and 30 s. The best models, presenting a minimal drop in performance at 10 s and 20 s compared to the reference 30 s interval, are highlighted.

This test applies the same image processing algorithm without modifications, regardless of the processing interval, simulating real-life scenarios with varying ECG signal availability. Specifically, ECHOView images for 10, 20, and 30 s were input into DNNs that had already been retrained or fine-tuned on 30 s images. Their AUROC on 30 s (Table 2) is compared to the 20 and 10 s intervals in Figure 9.

As expected, the 30 s analysis provides the highest accuracy (AUROC: 0.980–0.993 for retrained and 0.988–0.994 for fine-tuned DNNs). Shortening the interval to 20 s has a minor impact, with AUROC drops of up to 4% for retrained and 2% for fine-tuned DNNs. However, at 10 s, some models present substantial declines—up to 55% for retrained ResNet50V2 and 33% for fine-tuned ResNet50V2 and MobileNetV2 (α = 1, 1.4).

Nevertheless, some models remain robust at shorter analyses. Retrained MobileNetV2 (α = 0.35), EfficientNetB1, EfficientNetB2, and DenseNet121 show AUROC reductions of only 6–9%, while fine-tuned VGG16, EfficientNetV2B0, EfficientNetV2B1, and DenseNet-121, -169, -201 maintain stable performance with just a 4–8% decline.

#### 3.1.5. Noise Robustness over Full-Length Holter ECG Recordings

For real-life Holter ECG applications, AF detection models must be robust across full-length recordings, accounting for rhythm variations and noise from daily activities. This test included 30 complete recordings (24–72 h each) from the IRIDIA-AF database (records 137–166, Leads I and II) that were not part of the training or validation sets. All sequential 30 s ECG segments were extracted without overlap. No exclusion criteria were applied for noise or other events. Using the original IRIDIA-AF annotations, we analyzed a total of 213,516 ECHOView images, including 65,986 AF and 147,530 non-AF samples from Lead I and Lead II.

**Figure 10:**
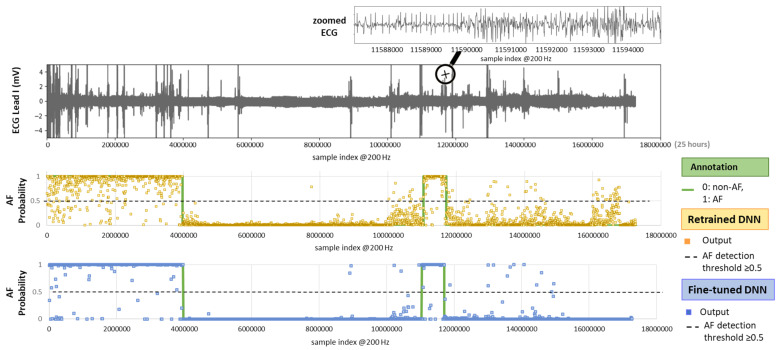
On **top**: 24 h Holter ECG recording (Lead I, record_157 from the IRIDIA-AF database) with a zoomed-in section for clearer visualization of the ECG waveform. On **bottom**: Original AF annotation together with AF probability outputs of the retrained and fine-tuned versions of a DNN (DenseNet121) computed for sequential, non-overlapping 30 s intervals across the entire recording.

Figure 10 illustrates the test setting with an example of a full-length 24 h Holter ECG recording. The ECG trace displays multiple spikes from extreme noise events, while the zoomed-in section highlights details of rhythm and noise transitions captured during routine monitoring. The AF prediction output of both retrained and fine-tuned versions of the DenseNet121 model is shown as multiple dots, each corresponding to the analysis of a sequential 30 s ECG segment. By applying an AF probability threshold of 0.5 to the DNN output, we can observe that the model’s decisions largely align with the AF annotations. However, the retrained DNN shows greater deviation, while the fine-tuned version demonstrates closer adherence to the annotations.

**Figure 11:**
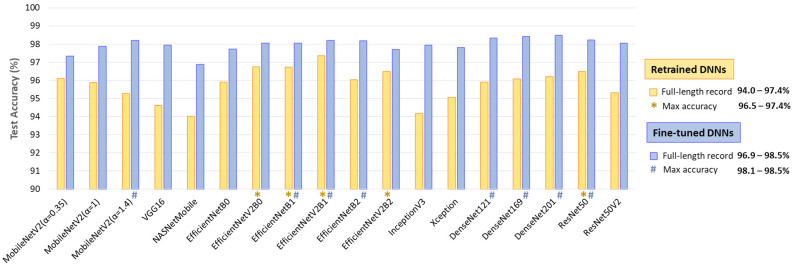
Test accuracy of 18 retrained and fine-tuned DNNs using ECHOView images generated through sequential 30 s analyses of 30 full-length records from the IRIDIA-AF database (records 137 to 166, Leads I and II) without excluding episodes affected by noise or other factors. The top-performing models with the highest accuracy are highlighted.

The results in Figure 11 indicate the test accuracy over the full-length Holter ECG recordings in the range of 94–97.4% for retrained DNNs and 96.9–98.5% for fine-tuned DNNs. Certain models demonstrate better noise and rhythm variability robustness in continuous monitoring, such as retrained EfficientNetV2B0, EfficientNetB1, EfficientNetV2B2, and ResNet50 with accuracy >96.5%; and fine-tuned MobileNetV2 (α = 1.4), EfficientNetB1, EfficientNetV2B1, EfficientNetB2, DenseNet-121, -169, -201, and ResNet50 with accuracy >98.1%.

#### 3.1.6. DNN Model Rankings Across All Tests

**Figure 12:**
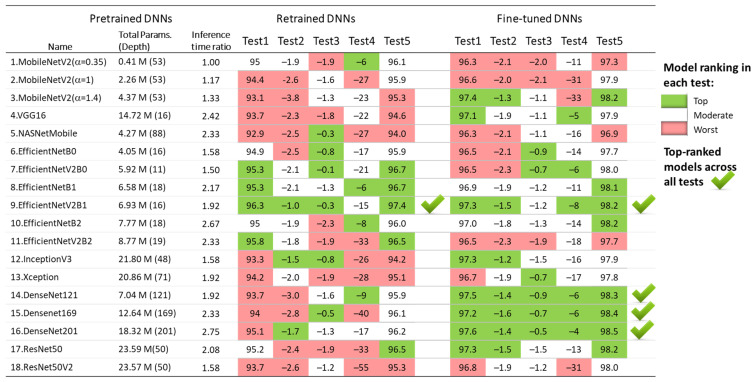
Ranking of 18 retrained and fine-tuned ImageNet models based on their AF detection performance in five tests: Test1 (test accuracy), Test2 (drop of test vs. validation accuracy), Test3 (drop of Lead II vs. Lead I accuracy), Test4 (drop of accuracy for 10 s vs. 30 s analysis), and Test5 (test accuracy over 30 full-length Holter ECG recordings).

The results of the five test concepts applied to the retrained and fine-tuned DNNs in Section 3.1.1, Section 3.1.2, Section 3.1.3, Section 3.1.4 and Section 3.1.5 are summarized in Figure 12. Although all models show competitive performance, some of them can be highlighted as the best in some tests, but the worst in others. For example, the fine-tuned MobileNetV2 (α = 1.4) presents one of the best test accuracies on the 30 s test set (Test 1) and full-length Holter recordings (Test 5), and it has a minimal drop of test vs. validation accuracy (Test 2); however, it shows moderate inter-lead stability (Test 3) and fails at a short analysis duration of 10 s (Test 4).

Since the aim of the transfer learning in this study is to underline the top-performing retrained and fine-tuned DNNs across all tests, we define the following criteria:

- Model that contains the maximum number of top-ranks (green cells in Figure 12);
- Model that has not achieved the worst rank in any of the five tests (red cells in Figure 12).

Based on these criteria, the underlined best models across all tests are both retrained and fine-tuned versions of EfficientNetV2B1 with four top-ranks, as well as the three fine-tuned DenseNet models (-121, -169, -201) with five top-ranks. For enhanced clarity and reproducibility, the architectures of the best performing models are visualized and explained in Supplementary Materials S1, according to the best practices for DNN models representation [80,81].

### 3.2. DNN Model Interpretability for AF Detection: Case Studies

This section presents case studies of representative AF and non-AF samples from the IRIFIA-AF test set, illustrating the output heatmaps from a top-performing DNN model (retrained EfficientNetV2B1) generated by GradCAM. Each heatmap is overlaid on its respective input ECHOView image (240 × 240 pixels) and aligned with the corresponding 30 s ECG signal to illustrate model interpretability.

#### 3.2.1. Sinus Rhythm: Correctly Detected Cases

**Figure 13:**
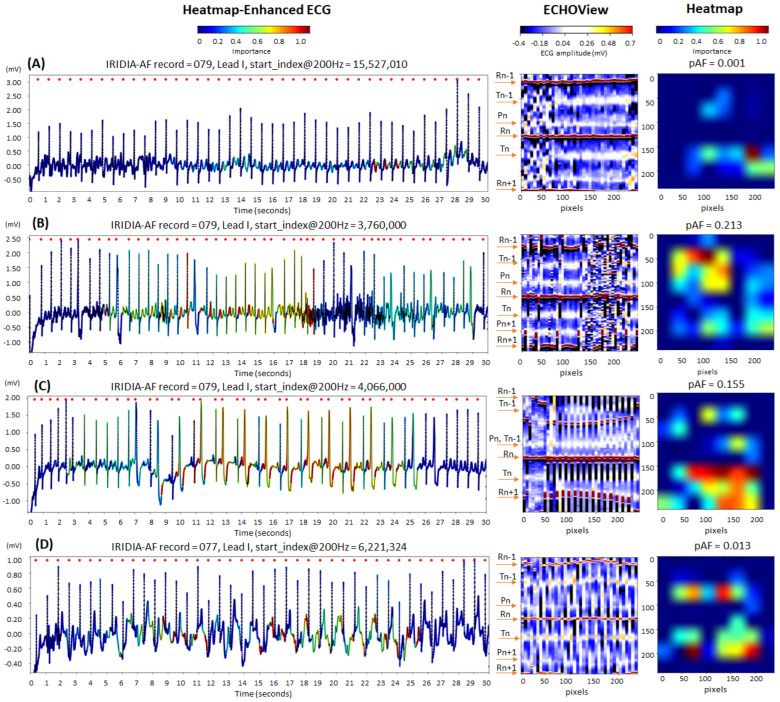
Four 30 s sinus rhythm ECG samples from the IRIDIA-AF database, illustrating the interpretability of the retrained EfficientNetV2B1 DNN through its output heatmap, generated from the input ECHOView image: (**A**) Sinus rhythm with EMG artifacts from 0 to 8 s; (**B**) sinus rhythm with ventricular extrasystoles and EMG artifacts from 17 to 27 s; (**C**) sinus rhythm with bigeminia from 7 to 27 s; and (**D**) sinus rhythm with motion-induced baseline drift. Red markers on the ECG trace indicate QRS positions. Markers on the ECHOView image denote key cardiac events: R-peaks of ventricular depolarization (Rn: current, Rn+1: next, Rn−1: previous), T-waves of ventricular repolarization (Tn: current, Tn−1: previous), and P-waves of atrial depolarization (Pn: current, Pn+1: next). The pAF values displayed above the heatmap represent the DNN output probability for atrial fibrillation (AF).

Figure 13 illustrates cases of correctly identified “non-AF” rhythms (pAF = 0.001–0.213), confirming normal sinus rhythm based on the predefined diagnostic criteria of our study. The ECG records displayed below show certain abnormalities commonly encountered in clinical practice, which can complicate rhythm determination and increase the risk of misdiagnosis:

- Electromyographic (EMG) artifacts: The heatmap-enhanced ECG shows that AF detection is not triggered during the intensive EMG artifacts (from 0 to 8 s in trace (A), and from 17 to 27 s in trace (B)), although the ECHOView image presents artifact-induced alterations.
- Motion artifacts: Although a motion-induced baseline drift in trace (D) persists throughout the entire recording and affects the entire ECHOView image, the DNN highlights only a few ECG segments resembling AF, primarily in the isoelectric line between the T and P waves, significantly altered by the noise.
- Multiple ventricular extrasystoles: The presented records show frequent extrasystoles, causing rhythm irregularity. Combined with the challenge of detecting P-waves due to recording artifacts, this makes the diagnosis of sinus rhythm more uncertain. In the presented cases, the DNN is not activated by the ventricular extrasystoles in trace (B). However, in trace (C), DNN is activated only after at least two bigeminy cycles, specifically in the region of the shortened RnRn+1 interval, including the entire extrasystole waveform. Nevertheless, these small activation segments on the heatmap do not affect the DNN’s final decision for non-AF.

#### 3.2.2. Atrial Fibrillation: Correctly Detected Cases

**Figure 14:**
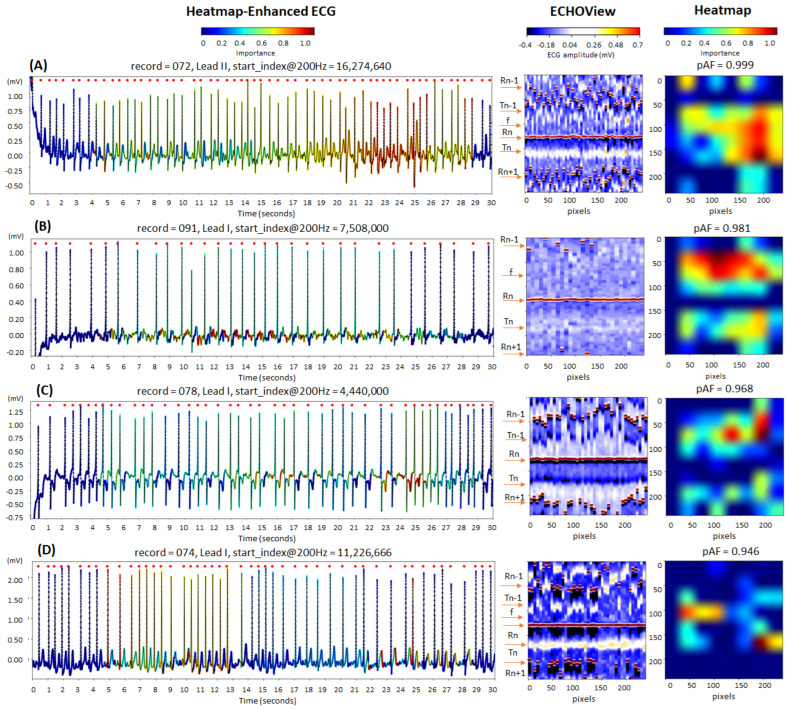
Four 30 s atrial fibrillation (AF) ECG samples from the IRIDIA-AF database, illustrating the interpretability of the retrained EfficientNetV2B1 DNN through its output heatmap, generated from the input ECHOView image: (**A**–**D**) Examples of atrial fibrillation rhythms from four patients. Red markers on the ECG trace indicate QRS positions. Markers on the ECHOView image denote key cardiac events: R-peaks of ventricular depolarization (Rn: current, Rn+1: next, Rn−1: previous), T-waves of ventricular repolarization (Tn: current, Tn−1: previous), and f-waves of atrial fibrillation (random white dots). The pAF values displayed above the heatmap represent the DNN output probability for AF.

The cardiac rhythm in the examples in Figure 14 is classified correctly as AF (pAF = 0.946–0.999), identifying the most informative ECG regions for the DNN:

- QRS complexes, corresponding to the most irregular and shortened RR-intervals in traces (A) and (D);
- Atrial fibrillatory f-waves, highlighted on the ECG signal near the isoelectric line in trace (B) and overlapping the T-waves in trace (C). The activated regions on the heatmap align with specific areas of the ECHOView image before and after the central QRS peak (Rn). This is an important notification in case (B), where low ECHOView contrast—due to long RR-intervals and low-amplitude f and T-waves—may impact the diagnostic interpretation.

#### 3.2.3. Atrial Fibrillation: Disputable Cases

**Figure 15:**
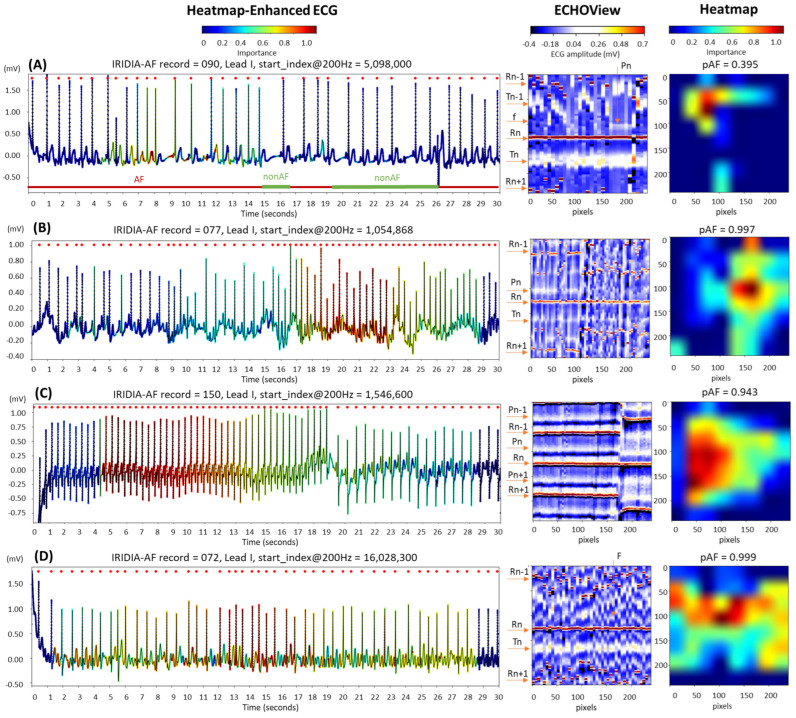
Four 30 s ECG samples from the IRIDIA-AF database, originally annotated as atrial fibrillation (AF) but later identified through visual evaluation by cardiologists as containing non-AF rhythms (either fully or partially): (**A**) Transition between AF and non-AF, as indicated below the ECG trace; (**B**) sinus rhythm disrupted by an artifact, followed by a rapid rhythm for which AF cannot be confirmed; (**C**) sinus tachycardia; and (**D**) atrial flutter. The figure demonstrates the ECG interpretability of the retrained EfficientNetV2B1 DNN using its output heatmap, generated from the input ECHOView image. Red markers on the ECG trace indicate QRS positions. Markers on the ECHOView image denote key cardiac events: R-peaks of ventricular depolarization (Rn: current, Rn+1: next, Rn−1: previous), T-waves of ventricular repolarization (Tn: current, Tn−1: previous), P-wave of atrial depolarization (Pn: current, Pn+1: next), f-waves of atrial fibrillation (random white dots), and F-waves of atrial flutter (white dots). The pAF values displayed above the heatmap represent the DNN output probability for AF.

The examples in Figure 15 are labeled as disputable because they were originally annotated as AF. However, after visual inspection by cardiologists, the rhythm was classified as non-AF, indicating sinus rhythm, either partially or throughout the entire 30 s ECG strip:

- In trace (A), a transition from AF to sinus rhythm is observed. The initial portion of the ECG (about 15 s) shows AF, with identifiable f-waves—key indicators for AF detection—visible either on the isoelectric line or superimposed on the T-waves, as highlighted by the heatmap. However, these regions are limited and insufficient for a definitive AF diagnosis (pAF = 0.395).
- Trace (B) begins with a sinus rhythm, which is disrupted at 16 s by an artifact and a rhythm change. The heatmap is activated immediately after the abrupt rhythm change, where shortened RR intervals are clearly visible in ECHOView. Although the activation corresponds to a relatively short ECG segment of just over 5 s, the DNN classifies it as strong enough for definitive AF detection (pAF = 0.997). However, visual inspection of the trace does not confirm AF, and another type of supraventricular tachycardia cannot be ruled out.
- Trace (C) presents an example of sinus tachycardia, as determined by cardiologists through visual inspection of the ECG record. The heatmap highlights a sustained rhythm with very short RR intervals, which was initially misclassified as AF by the algorithm (pAF = 0.943). However, this classification was later corrected when the RR intervals were prolonged. This example suggests that the DNN output may be influenced by heart rate, especially in rhythms with very short RR intervals.
- Trace (D) illustrates an example of atrial flutter, diagnosed through visual inspection by cardiologists. The heatmap shows strong activation in the region of F-waves in the middle part of the recording, where the rhythm is characterized by a rapid heart rate and pronounced RR interval variability. This activation led to the misclassification of atrial flutter as AF, with a high AF probability (pAF = 0.999). This suggests that the DNN was trained using mixed AF and atrial flutter annotations, which may explain why the algorithm misclassified atrial flutter as AF.

## 4. Discussion

### 4.1. DNN Model Interpretability for AF Detection

The timely and accurate detection of AF is critical from a clinical perspective, as the onset of this arrhythmia requires early initiation of anticoagulation to prevent cardioembolic events, primarily ischemic strokes [1,4,82,83]. The risk of cardioembolism is independent of the clinical phenotype of AF (newly diagnosed, paroxysmal, persistent, or permanent) and is largely determined by the patient’s age and associated risk factors, including chronic heart failure, hypertension, prior stroke or transient ischemic attack, embolism, vascular disease, and diabetes [1,83,84]. Clinical studies have shown that delaying anticoagulation beyond 12 to 24 h in AF episodes significantly increases stroke risk [1,4,82,83,84]. In patients with AF lasting more than 48 h without anticoagulation, approximately one in five develops an ischemic cerebrovascular event, which is often fatal [1]. More recent studies indicate that even brief, transient episodes of AF carry a substantial risk of cardioembolic events. Therefore, an early diagnosis is essential for ensuring a favorable intermediate and long-term prognosis in these patients [1,4,5,84,85].

This study validates that ImageNet DNNs applied to short-term ECHOView images through transfer learning can significantly enhance automated AF diagnosis. Such an application is valuable for the cost-effective evaluation of short or transient AF events in 24–72 h Holter ECG recordings. They often contain EMG and motion artifacts from daily activities, along with concomitant rhythm disorders like frequent extrasystoles or other types of supraventricular arrhythmias. It is crucial for the pretrained ImageNet DNNs to be precisely retrained to ensure an accurate diagnosis, thereby avoiding false AF identification, which could lead to unnecessary anticoagulation and associated bleeding complications [1,5,84,85]. Therefore, an important finding of our case study, which focuses on identifying ECG regions of interest for the DNN in diagnosing AF, is that the DNNs retrained with ECHOView image input are robust against EMG artifacts, motion artifacts, and arrhythmic events such as ventricular extrasystoles (Figure 13).

Representative AF cases highlight key regions for AF detection, including QRS complexes associated with irregular and shortened RR intervals, as well as f-waves near the isoelectric line or overlapping the T-waves (Figure 14).

The similarity between the heatmaps in Figure 14B and Figure 15D suggests that the DNN’s regions of interest for detecting f-waves in AF and F-waves in atrial flutter overlap. These regions appear to align with diagnostic areas near the isoelectric line, similar to those used by human experts. However, the atrial flutter sample in Figure 15D—annotated as AF and detected as AF with high probability (pAF = 0.999)—indicates that the DNN has not been trained to distinguish between f-waves and F-waves. This likely stems from the IRIDIA-AF training database, which may contain either an AF-class that groups both atrial fibrillation and atrial flutter samples or hold a small number of representative atrial flutter samples in the non-AF class. Notably, the presence and annotation of atrial flutter have not been communicated in [64].

Additionally, we highlight that the AF annotations in the IRIDIA-AF database should be interpreted with caution, given the other cases in Figure 15 that were identified by experts as transient AF, sinus rhythms, or tachycardias rather than persistent AF. These rhythms present a challenge for the DNN, which tends to classify them as AF due to the rapid and irregular heart rates. Nevertheless, the availability of this extensive Holter ECG database provides essential data for the effective deep transfer learning in this study.

### 4.2. Comparative Analysis of Transfer Learning DNNs for AF Detection

We benefit from deep transfer learning by using pretrained DNNs with up to 201 layers, which have already undergone extensive design, training, and optimization with large image datasets in the source domain. Modifying pretrained DNNs by changing only the top classification layer and retraining up to 2050 parameters, or fine-tuning up to 24 M parameters, requires fewer resources for adaptation to the AF classification framework. This allowed us to substantially expand the transfer learning study, adapting 18 pretrained DNNs from TensorFlow Keras applications [67], which have been demonstrated to be cost-effective in the source domain.

Although retraining and fine-tuning are applied to vastly different numbers of DNN architectures, depths, and model parameters (varying by millions), we do not observe a substantial difference in test performance. According to Table 2, fine-tuning improves test accuracy by 0.6–4.3%, with a greater effect on TPR (0.4–6.5%) and a smaller effect on TNR (0.6–2.8%). Furthermore, we do not observe a relation between the number of trainable parameters and test accuracy. For instance, retraining 513 (VGG16), 1025 (DenseNet121), and 2049 parameters (ResNet50V2) all yielded the same test accuracy of 93.7%. Similarly, fine-tuning 6.45 M parameters (EfficientNetV2B1) and 17.87–23.4 M parameters (DenseNet-121, -169, -201) resulted in very similar test accuracies of 97.2–97.6%. The same effect is observed in Figure 4, showing no relation between test accuracy and either computational cost or the total number of DNN parameters. For example, the small and fast MobileNetV2 (α = 1.4) model (4.37 M parameters, 1.33 inference time ratio) performs just as well as the larger and slower DenseNet201 model (18.32 M parameters, 2.75 inference time ratio).

The results in Table 2 indicate no substantial differences in test AF performance among the 18 implemented ImageNet DNN architectures. Test accuracy varies by up to 3.4% for retrained models (92.9–96.3%) and up to 1.3% for fine-tuned models (96.3–97.6%). To identify potential limitations of specific DNN architectures in processing ECHOView images across clinically relevant scenarios, we conducted four additional tests. These tests in Section 3.1.2, Section 3.1.3, Section 3.1.4 and Section 3.1.5 assess the impact of the non-stationary nature of Holter ECG signals across different patients (Figure 5), different ECG leads (Figure 7), different analysis durations (Figure 9), and robustness to noise sources and rhythm variations in continuous monitoring (Figure 11). The summary report in Figure 12 identifies the worst-ranked models, revealing a maximum inter-patient accuracy drop of 3.8% for the retrained MobileNetV2 (α = 1.4), a maximum performance drop of 2.3% for Lead II versus Lead I (retrained EfficientNetB2), a substantial 55% accuracy decrease for a 10 s analysis duration compared to 30 s (retrained ResNet50V2), and the lowest accuracy of 94% and 96.9% for long-term monitoring (retrained and fine-tuned NASNetMobile).

Figure 12 helped to finally underline the top-performing retrained and fine-tuned DNNs, which achieved the highest number of top-ranks and no worst ranks across all five tests, including the following:

- Retrained EfficientNetV2B1 has a 96.3% test accuracy with 2.1% inter-patient and 0.3% inter-lead drops; detects AF in shorter episodes with an accuracy drop of 0.6% (20 s) and 15% (10 s); presents 97.4% long-term monitoring accuracy.
- Fine-tuned EfficientNetV2B1 has a 97.3% test accuracy with 1.5% inter-patient and 1.2% inter-lead drops, detects AF in shorter episodes with an accuracy drop of 0.4% (20 s) and 8% (10 s), and presents 98.2% long-term monitoring accuracy.
- Three fine-tuned DenseNet models (121, -169, -201) have a 97.2–97.6% test accuracy with 1.4–1.6% inter-patient and 0.5–0.9% inter-lead drops; detect AF in shorter episodes with accuracy drop of 0.2–0.5% (20 s) and 4–6% (10 s); and present 98.3–98.5% long-term monitoring accuracy.

In resource-constrained implementations, EfficientNetV2B1 and DenseNet121 are preferable due to their lower computational demands, with an inference time ratio of 1.92 and approximately 7 million parameters. In contrast, DenseNet-169 and DenseNet-201 have higher inference time ratios of 2.33–2.75 and include 12.6–18.3 million parameters.

The reasons for the reliable performance of the models EfficientNetV2B1 and DenseNet (121, -169, -201) could be explained by referring to their architectures, which are visualized and discussed in detail in Supplementary Materials S1. In summary, the EfficientNetV2 family [72] embeds a compound scaling strategy that uniformly scales network width, depth, and resolution using fixed scaling coefficients, along with additional modifications to enhance training efficiency. Particularly, the architecture of the model EfficientNetV2B1 depicted in Figure S1 shows that it combines two Fused Mobile Inverted Bottleneck Convolutions (Fused-MBConv) with three MBConv stages. The integration of Fused-MBConv with regular convolutions in the early layers enhances computational efficiency, reduces memory overhead, and accelerates training, particularly on modern GPU-based hardware. Subsequent MBConv stages further optimize efficiency, relying on the inverted residual structure, which expands the input channels for greater feature extraction, applies depthwise convolution for spatial filtering, uses a squeeze-and-excitation attention mechanism to dynamically adjust channel importance, and projects back to a lower-dimensional representation for compact and efficient feature processing. Our findings are in agreement with other studies, which also report enhanced efficiency of the EfficientNet model family in various image processing applications [27,86,87,88,89].

The DenseNet (-121, -169, -201) models are part of the Densely Connected Convolutional Networks [75]. As illustrated in Figure S6, these models share a common structure comprising four dense blocks and three transition blocks. The main difference is the depth of the dense blocks and the number of feature maps, which increase with model complexity. The key structural element that makes DenseNet powerful in image processing is dense connectivity, where each layer within a dense block receives feature maps from all preceding layers. This provides feature reuse, strengthens gradient flow, and improves parameter efficiency, enabling deeper networks with fewer parameters. Combined with bottleneck layers for dimensionality reduction and transition blocks for downsampling, this architecture enhances feature representation, leading to superior performance in classification, segmentation, and other vision tasks [32,41,90,91,92].

This transfer learning study investigated pretrained models, relying on the optimization of their structural architectures and parameters in the source domain. Nevertheless, a promising future perspective for improvements is to create hybrid architectures by combining structural elements from the most successful models, such as EfficientNetB0, followed by a hybrid squeeze-and-excitation block with two parallel attention streams for feature refinement [93]. However, these structural enhancements would require careful architectural designs, optimizations, and training the model parameters from scratch with sufficiently large datasets.

### 4.3. Comparative Analysis with Published Studies

The top-performing retrained and fine-tuned DNNs for AF detection using 30 s ECHOView inputs from the IRIDIA-AF Holter ECG database can be compared to a few published studies with similar applications. Table 3 presents these studies, which use technology with the same principle as ECHOView for ECG-to-image transformation, namely the Electrocardiomatrix, and process the images by deep CNN models [18,19,20].

We found that the published CNN models in Table 3 were trained from scratch on limited ECG data. They report lower test accuracy (78.6–86.4%) compared to the accuracy achieved in this study (96.3–97.6%). For a fair comparison, however, it is important to consider key differences between studies, such as model complexity, analysis durations, databases, and output classes. In this context, the following dissimilarities between IRIDIA-AF [64], MITBIH-AF [94,95], MITBIH-Arrhythmia [95,96], and CinC Challenge 2017 [97] datasets could be outlined:

- ECG acquisition devices: The IRIDIA-AF dataset was recorded using Holter devices (200 Hz sampling rate, unspecified bandwidth), while ambulatory ECG recorders were used for the MIT-BIH-AF (250 Hz, 0.1–40 Hz) and MIT-BIH-Arrhythmia datasets (360 Hz, 0.1–100 Hz). The CinC Challenge 2017 dataset, in contrast, was collected using AliveCor ECG sensors (300 Hz, 0.5–40 Hz). Despite variations in sampling frequency and precision, the IRIDIA-AF, MIT-BIH-AF, and MIT-BIH-Arrhythmia datasets share similar acquisition conditions—recordings were taken from chest-mounted devices by healthcare professionals over extended periods (10–24 h) during daily activities. Conversely, the CinC Challenge 2017 dataset consists of short ECG recordings (9–61 s) captured via hand-held AliveCor sensors, with signals acoustically transmitted to a smartphone. This method is prone to acquisition errors, such as improper sensor placement, bad electrode contact, or inverted electrodes [97], potentially impacting AF detection accuracy as reported in [20].
- AF rhythm types: The IRIDIA-AF dataset explicitly includes only paroxysmal AF cases, excluding persistent and permanent AF. Similarly, the AF episodes in the MITBIH-AF dataset are described as “mostly paroxysmal”. However, the MIT-BIH Arrhythmia and CinC Challenge 2017 datasets do not provide specific details regarding AF types. The potential presence of multiple AF types (paroxysmal, persistent, and permanent) in these datasets could contribute to variability in detection performance and may have influenced the TPR reported in [20].
- Non-AF rhythm types: The IRIDIA-AF and MIT-BIH-AF datasets include ECG recordings from patients with at least one paroxysmal AF episode, without specific selections based on non-AF rhythm variety. In contrast, half of the recordings in the MITBIH-Arrhythmia dataset have been selected to include less common but clinically significant arrhythmias that would not be well-represented in a small random sample [96]. Similarly, the large patient cohort in the CinC Challenge 2017 dataset suggests a wide range of heart disease diagnoses, grouped under the annotation “Other rhythms”. The increased variability in non-AF rhythms within the MIT-BIH Arrhythmia and CinC Challenge 2017 datasets may contribute to the lower TNR observed in [19,20] compared to [18] and this study.
- Noise presence: The IRIDIA-AF dataset explicitly excludes ECG recordings with insufficient quality or excessive noise. We hypothesize that this quality-based selection process was based on observations from only segments of the Holter ECG recordings, as we observed significant noise consistently present throughout the entire 24–72 h duration of the records. In contrast, the CinC Challenge 2017 dataset includes a separate ECG class labeled “too noisy to be classified”. Therefore, the classification task is different, presenting one mixed class of AF and noise in this study versus two separate classes of AF and noise in [20]. Information on the presence of noise in MITBIH-AF and MITBIH-Arrhythmia datasets is not available, and relevant conclusions regarding noise impact cannot be derived.

Nevertheless, we believe that the use of pretrained DNNs by transfer learning in combination with the new large-scale IRIDIA-AF database with paroxysmal AF contributed to the effective training of powerful AF classification models with 7 M to 18 M parameters. The reliability of the database is determined by the large amount of ECG data collected in different ambulatory conditions, together with the wide demographic diversity in terms of gender, age, and risk of stroke CHADVASC score. Examples for such effective training with the IRIDIA-AF database of another large DNN (9 residual blocks with three convolutional layers in each) can be found in [64]. Using a similar duration of the single-lead raw ECG input (40 s), Gilon et al. [64] report comparable performance on a patient-level separated test set (TPR = 95.2%, TNR = 99.2%, Acc = 97.1%) vs. similar test-set policy in this study (TPR = 93.9–95.8%, TNR = 98.7–99.5%, Acc = 96.3–97.6%).

This is a research-oriented study, and we do not consider the regulatory aspects for real-world deployment of retrained and fine-tuned DNNs in medical devices [98]. The safety and performance of artificial intelligence and machine learning in software as a medical device are critical topics that necessitate validation by independent entities. Given the increased risk of overfitting, extensive validation is essential. Nevertheless, the explainability provided through heatmaps can contribute to regulatory evaluations. Furthermore, real-world applications should account for a broader range of rhythm classes beyond just atrial fibrillation.

## 5. Conclusions

This is one of the largest deep transfer learning studies for short-term single-lead AF detection in Holter ECG, which compares 18 cost-effective ImageNet DNNs (<24 M parameters) for their ability to adapt to a new image recognition domain using diagnostic ECHOView images. After retraining and fine-tuning, DNNs did not present a substantial difference in test performance, despite their different architectures. The achieved high AF detection accuracy of retrained (92.9–96.3%) and fine-tuned DNNs (96.3–97.6%) confirms that all architectures can effectively process ECHOView images. We do not observe a relation between the test accuracy and either computational cost or the number of trainable parameters.

Further tests, reproducing four clinically relevant scenarios, demonstrate strong generalizability for a few networks—retrained and fine-tuned EfficientNetV2B1, and fine-tuned DenseNet-121, -169, -201. A small accuracy drop across different patients (1–1.6%) indicates that these models capture generalizable ECG features, while remaining minimally sensitive to patient-specific variations. Their robust performance across both leads, with only a 0.3–1.2% difference, enables users to select the most convenient or least noisy lead for processing, while ensuring that AF detection remains accurate and unaffected by lead-specific variations. In real-world scenarios with varying ECG signal availability, the highlighted models trained on 30 s ECHOView images can detect AF in shorter episodes with a small accuracy drop (<0.6%) when reduced to 20 s, and a larger drop (4–15%) when reduced to 10 s. Moreover, rhythm variations and noise from daily activities evaluated in 30 full-length Holter ECG recordings (24–72 h each) were found not to affect the accuracy of the outlined models for 30 s analysis intervals (97.4–98.5%).

Using GradCAM, we gained meaningful insights into the decision-making process of the retrained EfficientNetV2B1, which focuses on ECG regions of interest that align with cardiologist interpretations. The presented non-AF examples demonstrate that DNNs retrained with ECHOView image input are robust against EMG artifacts, motion artifacts, and arrhythmic events such as ventricular extrasystoles. Representative AF cases highlight key detection regions, including QRS complexes associated with irregular and shortened RR intervals, as well as f-waves near the isoelectric line or overlapping the T-waves. Additionally, we observe that some rhythms with rapid and irregular heart rates present a challenge for the DNN, resulting in high-probability AF classification.

These findings emphasize the effectiveness of transfer learning in improving AF detection accuracy while maintaining computational efficiency, making it a superior alternative to training CNN models from scratch on limited datasets. Further studies adopting this approach should be conducted to detect AF in larger volumes of data, thereby validating the methodology. Additionally, the approach could be trained and tested in the context of more complex scenarios, involving multi-label classification of arrhythmias.

## Figures and Tables

**Table 1:** Sample size of the training, validation, and test datasets extracted from the IRIDIA-AF database using 30 s analysis intervals.

	Training	Validation	Test
Records	000–071	000–071	072–166
Non-AF samples (Lead I/Lead II)	3384/3384	1656/1656	2850/2850
AF samples (Lead I/Lead II)	3384/3384	1656/1656	2850/2850
Total samples	13,536	6624	11,400

**Table 2:** List of 18 pretrained DNNs used in this study and the test AF detection performance of their retrained and fine-tuned versions. D: Input image size (D × D × 3); TPR: true positive rate; TNR: true negative rate; Acc: accuracy; AUROC: area under the receiver operating characteristic curve; Depth: number of layers or stages in the architecture that might include different combinations of convolutional, depthwise convolutional, and fully connected layers; α: width multiplier, scaling the number of filters in each layer of MobileNetV2.

Pretrained DNNs				Retrained DNNs					Fine-Tuned DNNs				
Name	Input D	Total Params (Depth)	Inference Time Ratio	Trainable Params	TPR (%)	TNR (%)	Acc (%)	AUROC	Trainable Params	TPR (%)	TNR (%)	Acc (%)	AUROC
MobileNetV2 (α = 0.35) [68]	224	0.41 M (53)	1.00	1281	92.0	98.0	95.0	0.988	0.38 M	93.7	98.9	96.3	0.990
MobileNetV2 (α = 1) [68]	224	2.26 M (53)	1.17	1281	91.2	97.6	94.4	0.986	2.19 M	94.1	99.1	96.6	0.990
MobileNetV2 (α = 1.4) [68]	224	4.37 M (53)	1.33	1793	89.3	96.9	93.1	0.981	4.27 M	**95.4 #**	**99.5 #**	**97.4 #**	0.991
VGG16 [69]	224	14.72 M (16)	2.42	513	91.1	96.4	93.7	0.980	14.72 M	**95.1 #**	**99.2 #**	**97.1 #**	0.991
NASNetMobile [70]	224	4.27 M (88)	2.33	1057	88.2	97.5	92.9	0.980	4.20 M	93.4	99.1	96.3	0.992
EfficientNetB0 [71]	224	4.05 M (16)	1.58	1281	91.6	98.2	94.9	0.989	3.97 M	93.6	99.4	96.5	0.991
EfficientNetV2B0 [72]	224	5.92 M (11)	1.50	1281	92.2	98.5	95.3	0.991	5.78 M	94.0	99.1	96.5	0.994
EfficientNetB1 [71]	240	6.58 M (18)	2.17	1281	92.1	98.4	95.3	0.991	6.45 M	94.5	99.4	96.9	0.993
EfficientNetV2B1 [72]	240	6.93 M (16)	1.92	1281	93.9 *	98.7 *	96.3 *	0.993	6.79 M	**95.3 #**	**99.4 #**	**97.3 #**	0.994
EfficientNetB2 [71]	260	7.77 M (18)	2.67	1409	91.7	98.3	95.0	0.988	7.63 M	94.6	99.4	97.0	0.993
EfficientNetV2B2 [72]	260	8.77 M (19)	2.33	1409	93.3	98.3	95.8	0.993	8.61 M	93.7	99.3	96.5	0.993
InceptionV3 [73]	224	21.80 M (48)	1.58	2049	89.8	96.8	93.3	0.981	21.75 M	**95.2 #**	**99.4 #**	**97.3 #**	0.992
Xception [74]	224	20.86 M (71)	1.92	2049	91.2	97.3	94.2	0.986	20.75 M	94.4	99.0	96.7	0.988
DenseNet121 [75]	224	7.04 M (121)	1.92	1025	89.1	98.3	93.7	0.988	6.87 M	**95.6 #**	**99.3 #**	**97.5 #**	0.991
Densenet169 [75]	224	12.64 M (169)	2.33	1665	89.7	98.3	94.0	0.987	12.33 M	**95.1 #**	**99.4 #**	**97.2 #**	0.994
DenseNet201 [75]	224	18.32 M (201)	2.75	1921	91.6	98.7	95.1	0.991	17.87 M	**95.8 #**	**99.5 #**	**97.6 #**	0.989
ResNet50 [76]	224	23.59 M (50)	2.08	2049	92.1	98.4	95.2	0.990	23.48 M	**95.4 #**	**99.3 #**	**97.3 #**	0.992
ResNet50V2 [76]	224	23.57 M (50)	1.58	2049	89.8	97.6	93.7	0.986	23.48 M	94.3	99.3	96.8	0.992

* Bolded: The best performing retrained DNNs with Acc > 96%. # Bolded: The best performing fine-tuned DNNs with Acc > 97%.

**Table 3:** AF detection performance of the top-ranked transfer learning DNNs in this study compared to published studies, using CNN and similar to ECHOView images (Electrocardiomatrix) on public ECG databases. ECM: Electrocardiomatrix, TPR: true positive rate, TNR: true negative rate, Acc: accuracy.

Study	DNNs (Model Parameters)	Input (Analysis Interval)	Database	TPR, %	TNR, %	Acc, %
This study	Retrained EfficientNetV2B1 (6.93 M)	ECHOView images (30 s)	IRIDIA-AF	93.9	98.7	96.3
	Fine-tuned EfficientNetV2B1 (6.93 M)			95.3	99.4	97.3
	Fine-tuned DenseNet-121 (7.04 M)			95.6	99.3	97.5
	Fine-tuned DenseNet-169 (12.64 M)			95.1	99.4	97.2
	Fine-tuned DenseNet-201 (18.32 M)			95.8	99.5	97.6
Salinas-Martínez et al., 2020 [18]	CNN (11,257)	ECM images (10 beats + 3 s)	MITBIH-AF	78.3 *	91.2 *	85.1 *
Salinas-Martínez et al., 2021 [19]	CNN (10,217)	ECM images (10 beats + 3 s)	MITBIH-AF	79.7 ^&^	92.4 ^&^	86.4 ^&^
			MITBIH- Arrhythmia	91.7 ^&^	77.1 ^&^	78.6 ^&^
Lee et al., 2021 [20]	CNN (2.01 M)	ECM images (1 min)	CinC Challenge 2017	80.5 ^#^	85.6 ^#^	85.1 ^#^

* Mean value as reported in [18]. ^&^ Mean values calculated from the reported measurements for Lead I and Lead II in [19]. ^#^ Values for the AF detection performance are derived from a four-class confusion matrix (normal sinus rhythm, atrial fibrillation, other rhythms, noise) as presented in [20].

## Data Availability

The data of the large paroxysmal atrial fibrillation long-term electrocardiogram monitoring database (IRIDIA-AF) is publicly available on the Zenodoo website at https://zenodo.org/records/8405941, Version 1.0.1 (published 4 October 2023, https://doi.org/10.5281/zenodo.8186845), last accessed on 6 March 2025.

## Supplementary Materials

The following supporting information can be downloaded at https://www.mdpi.com/article/10.3390/diagnostics15070865/s1. Description of the top performing models in this study, including architectures of EfficientNetV2B1 and DenseNet (-121, -169, -201) (Supplementary Material S1).
