# Supplementary material for: Detection of Atrial Fibrillation in Holter ECG Recordings by ECHOView Images: A Deep Transfer Learning Study
Source: Diagnostics (Basel). 2025 Mar 28;15(7):865. doi: 10.3390/diagnostics15070865 (PMC11988734; doi:10.3390/diagnostics15070865)
Supplement: Supplementary file 1 [file diagnostics-15-00865-s001.zip › diagnostics-3544450-supplementary.pdf]

Article

Detection of Atrial Fibrillation in Holter ECG Recordings by ECHOView Images: A Deep Transfer Learning Study (Supplementary Material 1)

Vessela Krasteva [1], Todor Stoyanov [1], Stefan Naydenov [2], Ramun Schmid [3] and Irena Jekova [1*]

[1] Institute of Biophysics and Biomedical Engineering, Bulgarian Academy of Sciences, Acad. G. Bonchev Str. Bl. 105, 1113 Sofia, Bulgaria; vessika@biomed.bas.bg (V.K.), todor@biomed.bas.bg (T.S.), irena@biomed.bas.bg (I.J.)
[2] Department of Internal Diseases "Prof. St. Kirkovich", Medical University of Sofia, 1431 Sofia, Bulgaria; snaydenov@gmail.com (S.N.)
[3] Signal Processing, Schiller AG, Altgasse 68, CH-6341 Baar, Switzerland; ramun.schmid@schiller.ch (R.S)
* Correspondence: irena@biomed.bas.bg

Objectives: The purpose of this Supplementary Material is to present the architectures of the top-performing ImageNet deep neural networks (DNNs), according to the findings in the full-text of the study: "*Detection of Atrial Fibrillation in Holter ECG Recordings by ECHOView Images: A Deep Transfer Learning Study*". Specifically, it covers the models EfficientNetV2B1 and DenseNet (-121, -169, -201), which are integrated via TensorFlow Keras applications [1].

As this research employs transfer learning, preserving the original ImageNet architectures except for the replacement of the top (classification) layer, the provided further block diagrams of the DNN architectures are primarily for illustrative purposes. They visualize specific architectural details not explicitly documented in the official publications [2,3,4], thereby aiding researchers to understand the network's internal information pathways. Additionally, these block diagrams enhance transparency by summarizing the DNN architectures exactly as implemented, with ECHOView images as input and a binary classification output for atrial fibrillation (AF) detection.

1. Architecture of EfficientNetV2B1

EfficientNetV2B1 is a convolutional neural network (CNN) architecture that belongs to the EfficientNetV2 family [3]. These models are designed to achieve better performance and faster training speeds compared to their predecessors, the EfficientNetV1 models [2] by using a combination of architectural innovations, which are further disclosed. The flow diagram of the embedded EfficientNetV2B1 model is shown in Figure S1, including the following structural blocks:

1. Input Image

The ECHOView input image shape is 240x240x3, which is the default setting to initialize the model with pre-trained ImageNet weights and biases (6,931,124 parameters).

2. Stem Block

The Stem block includes the initial sequence of convolutional operations that reduces the spatial dimensions while increasing the number of feature maps. This provides efficient processing of input data before passing them to deeper network layers. As shown in Figure S2, EfficientNetV2B1 employs a stem convolution with a compact 3×3 kernel and a stride of 2×2, effectively reducing image dimensions by half while moderately expanding

the number of channels from 3 to 32. A Batch Normalization (BN) layer is applied to stabilize training and speed up convergence. The 'Swish activation function ('silu') is applied to introduce non-linearity and improve gradient flow

3. Block 1

Block 1 is a residual convolutional block with two standard 3×3 convolutions, each followed by batch normalization and Swish activation (Figure S2). It uses a residual connection, following a ResNet-style structure for improved gradient flow and training efficiency. As shown in Figure S2, this block maintains the spatial dimensions while reducing the number of channels from 32 to 16, optimizing feature extraction in the early stages of the network.

4. Blocks 2 and 3

Each block consists of three Fused Mobile Inverted Bottleneck Convolutions (Fused-MBConv), designed by following the principles in Figure S1 (right) and implemented according to the detailed flowcharts in Figure S3. Fused-MBConv starts with a standard 3×3 convolution with a stride of 2×2 or 1×1 for downsampling or maintaining dimensions, while expanding the number of channels from 16 to 192 in subsequent blocks. Further, a projection 1×1 convolution projects back to a smaller number of channels (32 or 48). Final BN ensures stability before the residual connection "Add", which just improves gradient flow. Note that Squeeze-and-Excitation (SE) block is not present, although shown in [3].

5. Blocks 4, 5 and 6

Blocks 4, 5 and 6 consist of 4, 6, and 9 MBConv stages, respectively, following the principle in Figure S1 (right) and implemented according to the detailed flowcharts in Figure S4. Each MBConv block begins with 1x1 convolution, which expands the number of channels from 192 to 1152 in subsequent stages. Then the depthwise 3x3 convolution applies spatial filtering with stride 2, reducing spatial dimensions. Next, the Squeeze-and-Excitation (SE) attention mechanism enhances feature representations by: (i) Global Average Pooling to compute spatial feature statistics; (ii) 1x1 convolution to reduce channels to 12, 24, 28, or 48, depending on the MBConv stage; (iii) 1x1 convolution to restore the channel count to match the MBConv input (excitation phase); (iv) multiplication that applies attention to the depthwise convolution output. The final projection stage involves 1x1 convolution to reduce channels to 96, 112 or 192 in subsequent blocks, followed by BN to normalize the final output.

6. Top Block

The top block serves as the transition from the feature extraction backbone to the classification head. According to the functional representation in Figure S5, it implements 1x1 convolution, increasing the number of feature channels to 1280. Then BN normalizes the convolutional output across channels, helping to stabilize training and improve generalization. Then a smooth non-linearity by the Swish activation improves gradient flow. Finally, Global Average Pooling computes the spatial average of the feature maps, providing a reduced representation in single 1280-dimensional vector, summarizing the extracted features.

7. Output Block

The output block is the classification stage, which processes the final feature vector and outputs a single prediction score. According to the flow diagram in Figure S5, the classification uses the fully connected (Dense) layer with 1 unit to solve a binary classification task. Then, applies sigmoid activation to convert the raw score into a probability (0 to 1 range). The output represents the probability of the input belonging to the positive class (AF).

In conclusion, the EfficientNetV2B1 architecture benefits from a good balance between Fused-MBConv and MBConv stages, combining their strengths for greater efficiency. In

the early stages, the use of Fused-MBConv with regular convolutions has been shown to provide significant advantages over the original MBConv with depthwise convolutions used in the first EfficientNet version [3]. These benefits include: improved computational efficiency, lower memory overhead, and faster training, particularly on modern GPU-based hardware. Meanwhile, the three MBConv blocks further improve efficiency, relying on the inverted residual structure, which expands the input channels for greater feature extraction, applies depthwise convolution for spatial filtering, uses an SE module for adaptive channel weighting, and projects back to a lower-dimensional representation for compact and efficient feature processing.

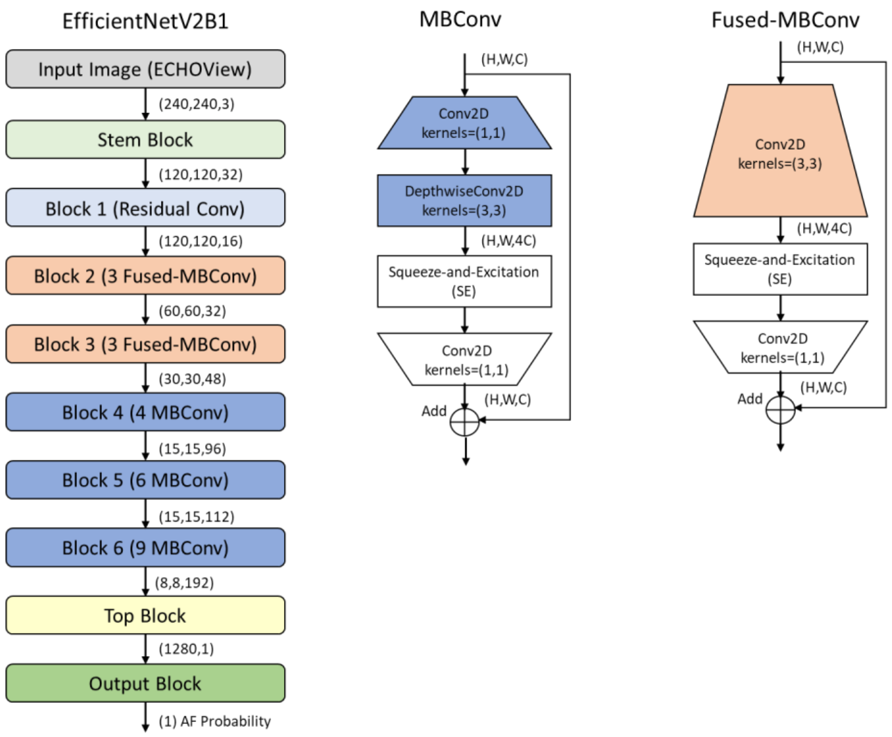

Figure S1. On Left: Flow diagram of EfficientNetV2B1 embedded in this study, using ECHOView images as input and a binary classification output for atrial fibrillation (AF) detection. **On Right:** Flow diagrams of two basic processing blocks: MBConv (Mobile Inverted Bottleneck Convolution) and Fused-MBConv, showing the original design principle as disclosed in [3].

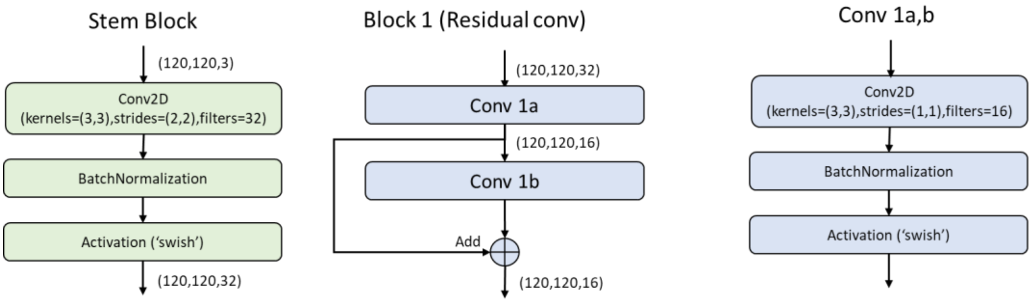

Figure S2. Structural details of EfficientNetV2B1: Stem block (left) and Residual Block 1 with two convolutional layers Conv 1a,b (right).

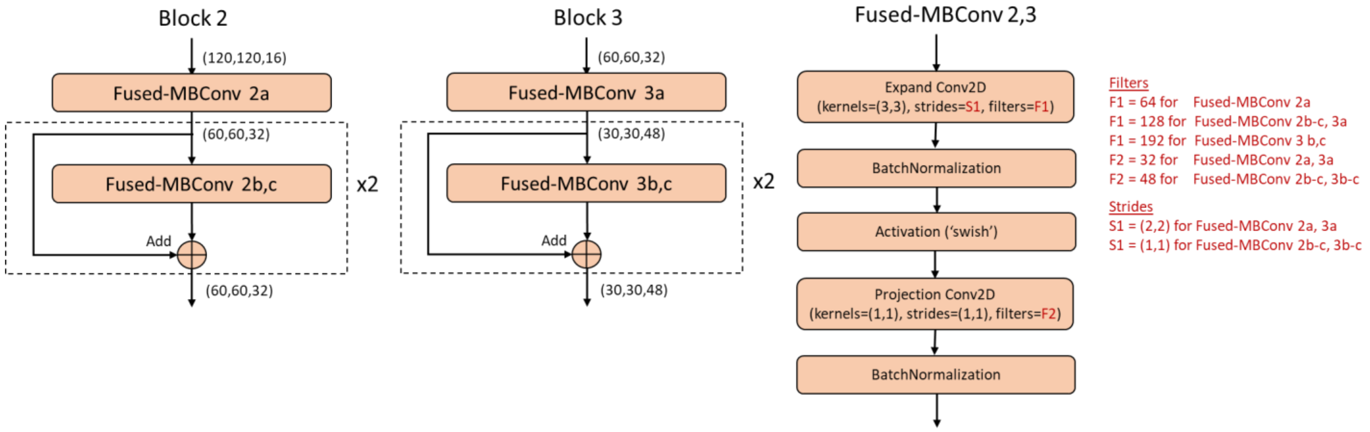

Figure S3. Structural details of EfficientNetV2B1: Block 2 and Block 3 (left) and the embedded Fused-MBConv operations (right). Fused-MBConv: Fused-Mobile Inverted Bottleneck Convolution. Note that the Fused-MBConv in EfficientNetV2B1 does not implement the Squeeze-and-Excitation block disclosed in the original design principle [3] and reproduced in Figure S1 (right).

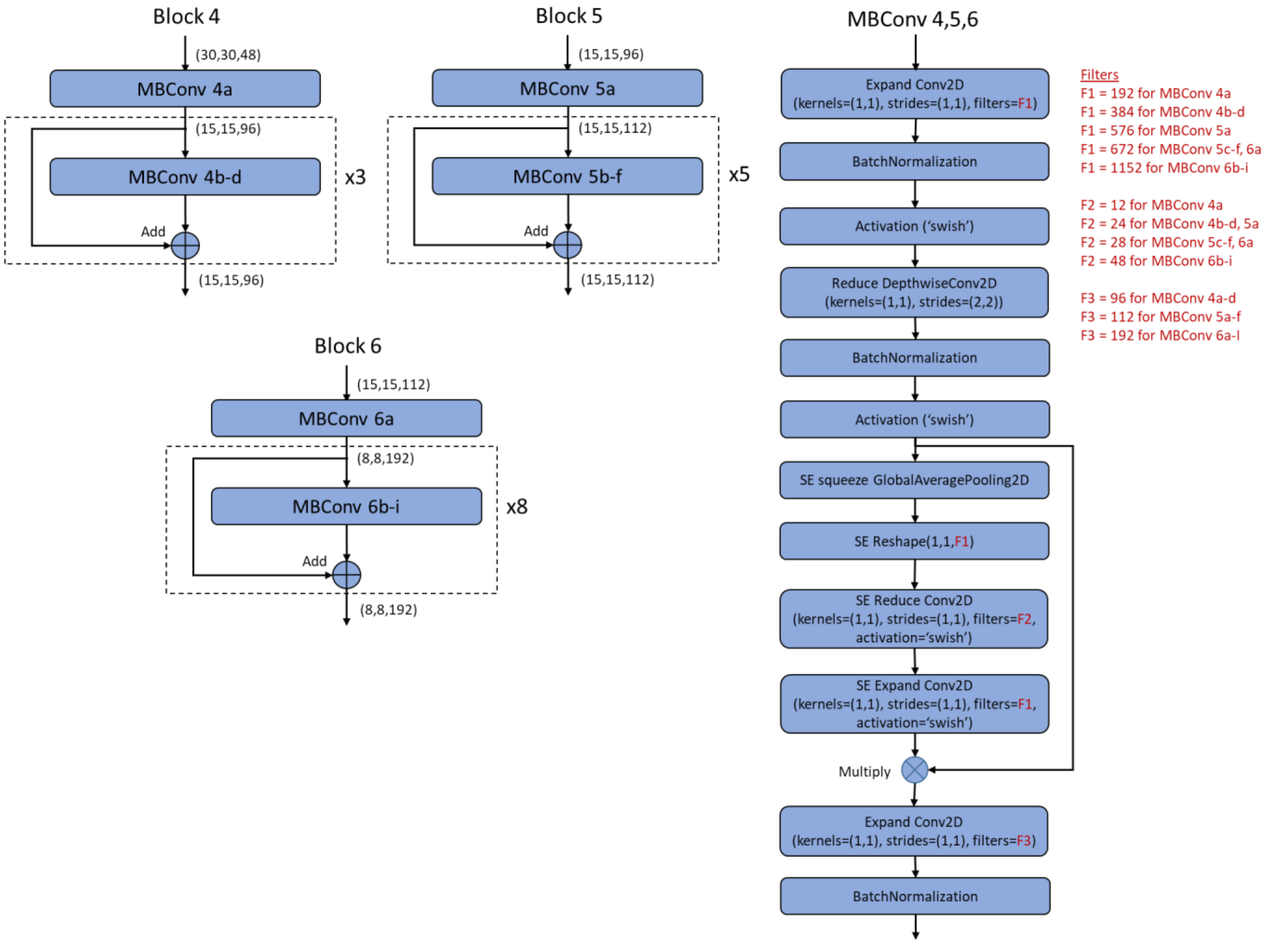

Figure S4. Structural details of EfficientNetV2B1: Block 4, Block 5 and Block 6 (left) and the embedded MBConv operations (right). SE: Squeeze-and-Excitation. MBConv: Mobile Inverted Bottleneck Convolution.

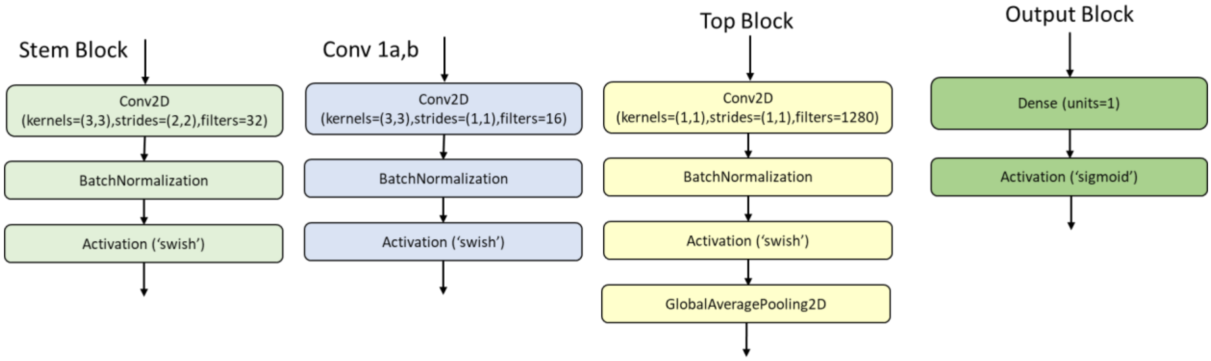

Figure S5. Structural details of EfficientNetV2B1: Top block (left) and Output block (right), modified for the binary classification of atrial fibrillation.

1. Architecture of DenseNet (-121, -169, -201)

DenseNet (-121, -169, -201) models belong to the Densely Connected Convolutional Networks [4]. The dense connectivity means that each convolutional layer receives inputs from preceding layers, shown to strengthen gradient flow and feature reuse. Figure S6 illustrates the flow diagrams of the networks implemented in this study, highlighting the structural differences among DenseNet-121 (7,038,529 parameters), DenseNet-169 (12,644,545 parameters), and DenseNet-201 (18,323,905 parameters). These variants differ in depth and feature map dimensions, providing a trade-off between accuracy and computational efficiency based on task requirements. Analysis of the DenseNets follows.

1. Input Image

The input image shape is 224x224x3, which is the default setting to initialize the models with pre-trained ImageNet weights and biases.

2. Input Block

The input block, illustrated in Figure S7, processes the input image and extracts initial low-level features through a sequence of operations. First, ZeroPadding2D adds a 3-pixel padding around the image to maintain spatial dimensions before convolution. Next, a 7×7 convolution with 64 filters and a stride of 2 reduces spatial dimensions while capturing essential low-level patterns. This is followed by BN and ReLU activation, which enhance training stability, accelerate convergence, and introduce non-linearity for better feature extraction. Another ZeroPadding2D layer then adds a 1-pixel padding before pooling to retain border information. Finally, a 3×3 max pooling operation with a stride of 2 further reduces spatial dimensions while preserving the most significant features.

3. Dense Block

The key element of the architecture is the dense block, whose functionality is illustrated in Figure S7. First, BN and ReLU activation normalize feature maps from the previous layer, stabilize training, improve convergence, and introduce non-linearity to enhance feature extraction. Next, a bottleneck layer with a 1×1 convolution and 128 filters reduces computational complexity and refines feature selection before applying a spatial transformation. This is followed by another round of BN and ReLU activation, providing the same benefits as before. Subsequently, a 3×3 convolution with 32 filters extracts local spatial features while maintaining resolution. Finally, these newly extracted features are concatenated with the original input features, establishing dense connectivity. This mechanism ensures that subsequent layers receive a richer set of features from all preceding layers, enhancing learning efficiency and gradient propagation.

4. Transition Block

The transition block in DenseNet serves as a bridge between dense blocks, reducing the number of feature maps and controlling model complexity while ensuring smooth

information flow. The functionality of the transition block is illustrated in Figure S7. First, BN and ReLU activation normalize feature maps from the previous dense block, introduce non-linearity, enhance feature representation, and improve gradient flow. Next, a bottleneck layer with a 1×1 convolution and 128 to 869 filters in sequential blocks reduces feature map dimensionality and computational cost while preserving critical information. This compression step limits the number of parameters and mitigates redundancy. Finally, average pooling with a 2×2 pool size and a stride of 2 downsamples spatial dimensions, enabling deeper layers to process progressively more abstract features. Unlike max pooling, average pooling smooths feature maps, providing a more general representation and reducing the risk of overfitting.

5. Top Block

The top block is the final stage of feature extraction before classification. It refines high-level representations from dense and transition blocks and prepares feature maps for decision-making. As shown in Figure S7, BN stabilizes activations and improves convergence. A ReLu layer introduces non-linearity, enhancing feature representation. Finally, Global Average Pooling replaces fully connected layers by averaging each feature map, reducing spatial dimensions to 1×1 and summarizing features into a compact vector (e.g., 1024 for DenseNet-121, 1280 for DenseNet-169,-201). This minimizes parameters and mitigates overfitting.

6. Output Block

The output block is the classification stage, which processes the final feature vector and outputs a single prediction score, using the fully connected (Dense) layer with 1 unit to solve a binary classification task (Figure S7). Then, applies sigmoid activation to convert the raw score into a probability (0 to 1 range). The output represents the probability of the input belonging to the positive class (AF).

As shown in Figure S6, all three DenseNet models (-121, -169, -201) share the same overall structure, consisting of four dense blocks and three transition blocks. The primary differences are: (i) the number of layers per dense block, which determines the total network depth (Table S1); (ii) the number of feature maps at Transition Block 3 and Dense Block 4, which increases cumulatively based on the growth rate and depth of previous layers (Table S2).

Table S1. Number of Layers per Dense Block.

Model	Dense Block 1	Dense Block 2	Dense Block 3	Dense Block 4	Total Layers
DenseNet-121	6	12	24	16	121
DenseNet-169	6	12	32	32	169
DenseNet-201	6	12	48	32	201

Table S2. Feature Maps at Transition Layers and Block 4.

Model	Transition Block 1	Transition Block 2	Transition Block 3	Block 4 Output
DenseNet-121	256 → 128	512 → 256	1024 → 512	1024
DenseNet-169	256 → 128	512 → 256	1280 → 640	1664
DenseNet-201	256 → 128	512 → 256	1792 → 896	1920

In conclusion, the key structural element that makes DenseNet powerful in image processing is the dense connectivity, where each layer within a Dense Block receives feature maps from all preceding layers. This provides feature reuse, strengthens gradient

flow, and improves parameter efficiency, enabling deeper networks with fewer parameters. Combined with bottleneck layers for dimensionality reduction and transition blocks for downsampling, this architecture enhances feature representation, leading to superior performance in classification, segmentation, and other vision tasks.

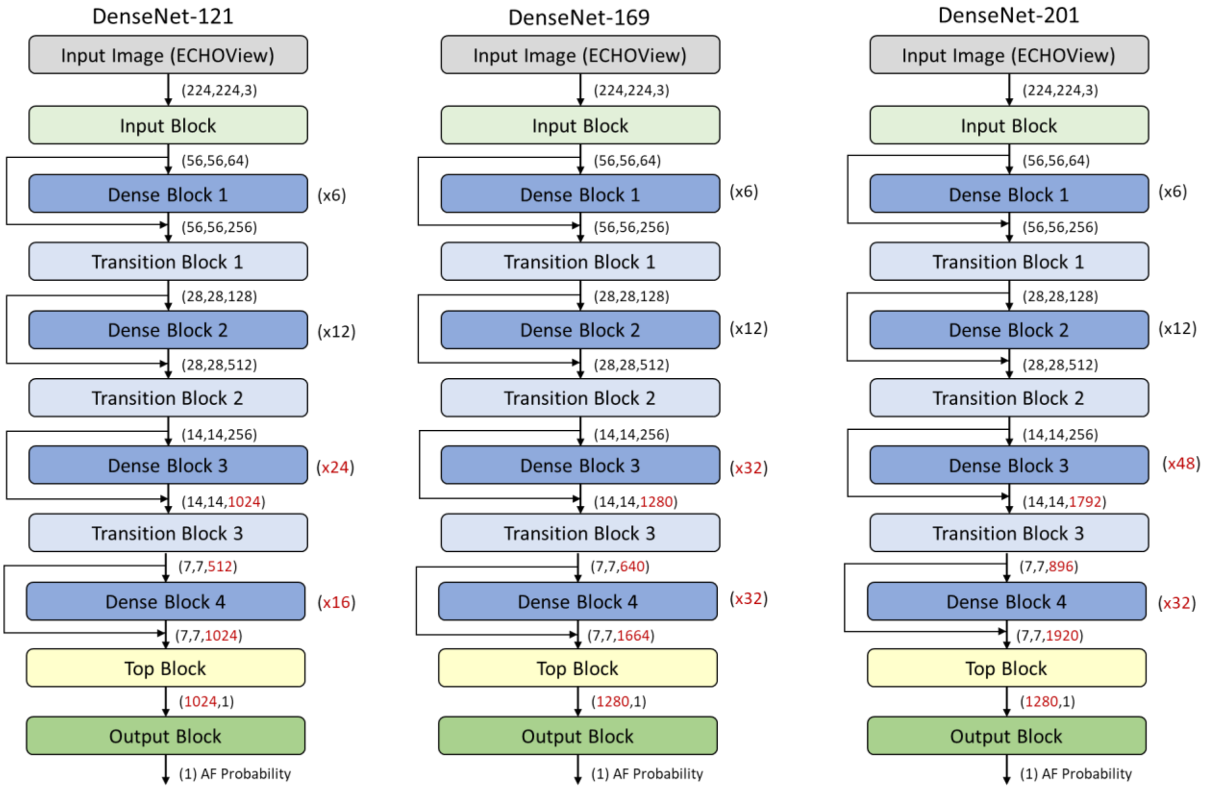

Figure S6. Flow diagrams of three DenseNet architectures embedded in this study, denoted as Densenet-121, DenseNet-169, and Densenet-201. They use ECHOView images as input and provide binary classification output for atrial fibrillation (AF) detection. The red text highlights the differences between different architectures, which share similar structural blocks.

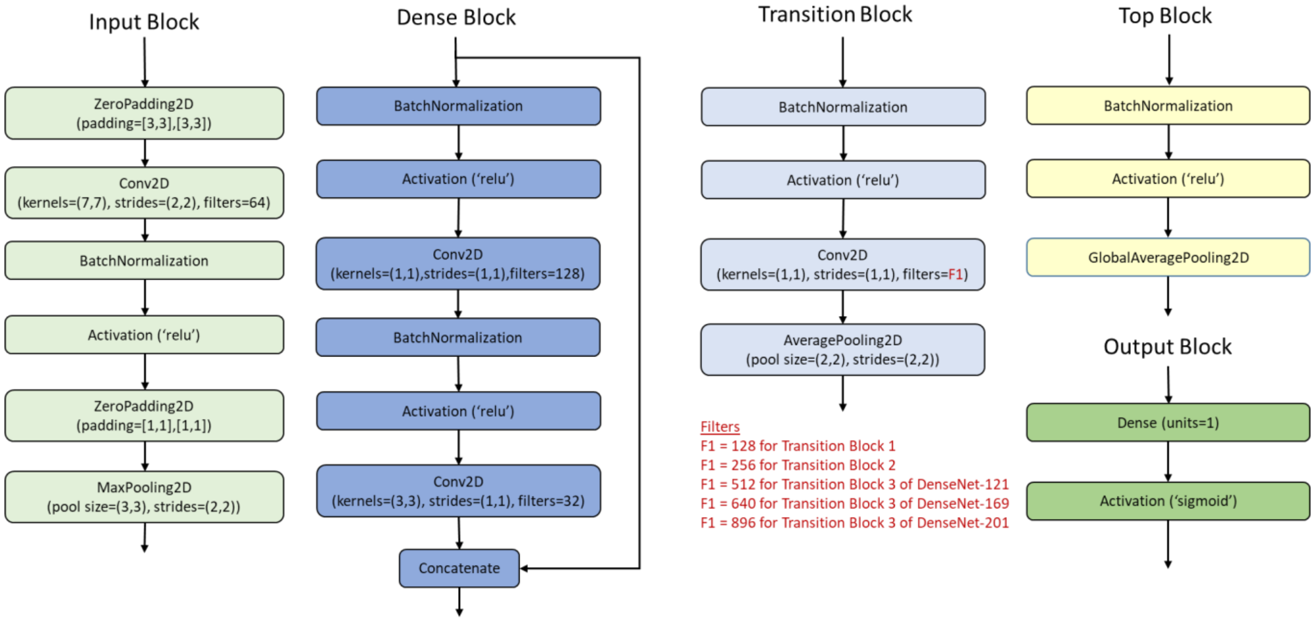

Figure S7. Structural details of DenseNet blocks.

References

1. Keras Applications. 2025. Available online: https://keras.io/api/applications/ (accessed on 05 March 2025).
2. Tan, M.; Le, Q.V. EfficientNet: Rethinking model scaling for convolutional neural networks, In *Proceedings of the 36th International Conference on Machine Learning, ICML* Long Beach, 9-15 June 2019, pp. 6105–6114. http://proceedings.mlr.press/v97/tan19a.html.
3. Tan, M.; Le, Q.V. EfficientNetV2: Smaller Models and Faster Training, arXiv:2104.00298v3, 2021, pp. 1–11, https://doi.org/10.48550/arXiv.2104.00298.
4. Huang, G.; Liu, Z.; Van Der Maaten, L.; Weinberger, K.Q. Densely Connected Convolutional Networks, In *Proc. IEEE Conference on Computer Vision and Pattern Recognition (CVPR),* Honolulu, HI, USA, 21-26 July 2017, pp. 2261–2269, doi: 10.1109/CVPR.2017.243.

Disclaimer/Publisher's Note: The statements, opinions and data contained in all publications are solely those of the individual author(s) and contributor(s) and not of MDPI and/or the editor(s). MDPI and/or the editor(s) disclaim responsibility for any injury to people or property resulting from any ideas, methods, instructions or products referred to in the content.
